# Comparative transcriptome analysis of isonuclear-alloplasmic lines unmask key transcription factor genes and metabolic pathways involved in sterility of maize CMS-C

**DOI:** 10.7717/peerj.3408

**Published:** 2017-05-30

**Authors:** Chuan Li, Zhuofan Zhao, Yongming Liu, Bing Liang, Shuxian Guan, Hai Lan, Jing Wang, Yanli Lu, Moju Cao

**Affiliations:** 1 Maize Research Institute, Sichuan Agricultural University, Chengdu, Sichuan, P.R. China; 2 Key Laboratory of Biology and Genetic Improvement of Maize in Southwest Region, Ministry of Agriculture, Chengdu, Sichuan, P.R. China

**Keywords:** Cytoplasmic male sterility, RNA-seq, Differentially expressed gene, Transcription factor, Metabolic pathway, Maize

## Abstract

Although C-type cytoplasmic male sterility (CMS-C) is one of the most attractive tools for maize hybrid seed production, the detailed regulation network of the male sterility remains unclear. In order to identify the CMS-C sterility associated genes and/or pathways, the comparison of the transcriptomes between the CMS-C line C48-2 and its isonuclear-alloplasmic maintainer line N48-2 at pollen mother cell stage (PS), an early development stage of microspore, and mononuclear stage (MS), an abortive stage of microspore, were analyzed. 2,069 differentially expressed genes (DEGs) between the two stages were detected and thought to be essential for the spikelet development of N48-2. 453 of the 2,069 DEGs were differentially expressed at MS stage between the two lines and thought to be participated in the process or the causes of microspore abortion. Among the 453 DEGs, 385 (84.99%) genes were down-regulated and only 68 (15.01%) genes were up-regulated in C48-2 at MS stage. The dramatic decreased expression of the four DEGs encoding MYB transcription factors and the DEGs involved in “polyamine metabolic process”, “Cutin, suberine and wax biosynthesis”, “Fatty acid elongation”, “Biosynthesis of unsaturated fatty acids” and “Proline metabolism” might play an important role in the sterility of C48-2. This study will point out some directions for detailed molecular analysis and better understanding of sterility of CMS-C in maize.

## Introduction

Plant cytoplasmic male sterility (CMS), a maternally inherited trait that resulted in the inability to produce functional pollen, is on the account of the inability to establish a coordinated interaction between the organellar and nuclear genomes (Fujii & Toriyama, 2008). Many of these defects can be restored by nuclear-encoded fertility restorer genes known as restorer-of-fertility (*Rf*) (Chase, 2007). CMS-Rf system circumvents the time-consuming and costly emasculation work in the production of hybrid seeds, thereby encouraging the system to generate *F*_1_ progenies in different field crops, such as rice, sorghum, sunflower and so on (Bohra et al., 2016). Maize male-sterile cytotype has been classified into three major groups, i.e., T (Texas), S (USDA), and C (Charrua), by their response to specific lines (Beckett, 1971). T cytotype is no longer widely used commercially in hybrid seed production due to its vulnerability to a fungal pathogen *Bipolaris* (*Helminthosporium*) *maydis* race T that causes southern corn leaf blight (Beckett, 1971; Wise et al., 1999). The commercial utilization of S cytotype is also negatively affected by the unstable fertility of CMS (Weider et al., 2009). CMS-C is today the most widely applied type of CMS for the production of hybrid seeds in maize due to the resistance to southern corn leaf blight and the stability of sterility (Sofi, Rather & Wani, 2007).

The analysis on molecular basis of maize CMS-C has a long history. Forde & Leaver (1980) previously demonstrated that there are differences in the mitochondrial polypeptides synthesis between the C and T or normal cytotype, but the functions of these polypeptides were not identified. Three chimeric genes (*atp9*-C, *cox II*-C, and *atp6*-C) that uniquely associated with the maize C cytotype were founded (Dewey, Timothy & Levings, 1991). From the aspect of whole mitochondrial genome, some potentially functional genes and open reading frames (ORFs) including the CMS associated chimeric ORFs had been identified (Allen et al., 2007). The evidence of how these genes and/or ORFs results in male sterility have not been obtained. At the transcriptome level, the CMS cytotype affecting the globe gene expression patterns were analyzed and some potential key genes and metabolism pathways associated with CMS were discussed in cotton (Yang, Han & Huang, 2014), soybean (Li et al., 2015), rice (Fujii, Komatsu & Toriyama, 2007; Hu et al., 2016), radish (Mei, Liu & Wang, 2016) and welsh onion (Liu et al., 2016). But the relationships between the maize C cytotype and the CMS remain unknown.

Chase (2007) suggested that isonuclear-alloplasmic CMS lines provide a window for the insights of cytoplasm-nucleus communication and is an ideal model for discovering the genes that are essential to pollen sterility. Here, genome-wide comparison of the transcriptome at different microspore development stages between the CMS-C line C48-2 and its isonuclear-alloplasmic maintainer line N48-2 were analyzed using RNA-seq. It will provide information on how the C cytotype affects the nuclear gene expression, and promote the elucidation of the genetic components and retrograde response pathways possibly involved in the sterility of CMS-C, then assist the utilization of CMS-C in maize hybrid breeding.

## Materials and Methods

### Plant materials

The maize CMS-C line C48-2 (C means the C cytotype) was obtained by successive backcrossing using N48-2 (N means the normal cytotype) as the male donor for more than eight generations (Fig. S1A). The two lines were cultivated in the same experimental plot in Sichuan Agriculture University (Chengdu, Sichuan Province, China). Three biological replicates of each line were planted at every two days, and each replicate was planted in three-row plots with 42 individual plants. Maize inflorescences are composed of spikelets which contain two florets, an upper and a lower one. The anthers in the upper floret develop 2–3 days ahead of the 3 anthers in the lower floret (Hsu, Huang & Peterson, 1988). In order to reconcile this developmental discrepancy, the lower florets were discarded and the rest of the spikelet were used for sample preparation.

### Samples preparation for qRT-PCR of transcriptional factors

Upper florets at different development stages were crushed by dissecting needles and dyed with carbol fuchsin stains, then observed by optical microscope (Leica DM1000) (Fig. S1C). Spikelets of ten independent plants at different microspore developmental stages, i.e., pollen mother cell stage (PS), dyad stage (DS), tetrad stage (TS) and mononuclear stage (MS), were collected and pooled respectively, and snap-frozen in liquid nitrogen and kept at −80 °C for RNA extraction.

### Samples preparation for RNA-seq

The spikelets at PS stage and MS stage were sampled and pooled from ten individual tassels of each plot respectively, and snap-frozen in liquid nitrogen and kept at −80 °C for RNA extraction. Three biological replicate samples were collected respectively and used for RNA-seq.

### RNA extraction

Total RNA was isolated using TRizol kit (invitrogen, USA) and purified using mRNA purification kit (Promega, USA) following the manufacturer’s protocol. An Ultra-microspectrophotometer NanoDrop 2000 (Thermo Fisher Scientific, USA) was used to detect total RNA concentration and purity.

### cDNA library construction and RNA-seq

The mRNA-enriched RNAs were broken into short pieces with the fragmentation buffer. The short pieces and random hexamer primers were used for the synthesis of the first strands of cDNAs. The second-strand cDNAs were synthesized using the Second Strand Synthesis Reaction Buffer and the Second Strand Synthesis Enzyme Mix from NEBNext^®^ Ultra™ RNA Library Prep Kit for Illumina (NEB, USA). Short fragments were purified with AMPure XP Beads (Agencourt, USA) for end repair and tailing A. A-tailed fragments were mixed with NEBNext Adaptor and Blunt/TA Ligase Master Mix for adaptor ligation. Finally, the sequencing libraries were constructed following PCR amplification with these adaptor-ligated fragments. Agilent 2100 Bioanalyzer and ABI StepOnePlus™ Real-Time PCR System were used for the quantification of the cDNA library. Paired-end sequencing was performed on the Illumina HiSeq 4000 sequencing platform. RNA-seq were completed by Beijing Annoroad Gene Technology Co., LTD (China).

### Read processing and mapping

All sequencing data were first processed by trimming adapter sequences, removing higher N rate (>10%) sequences and filtering low-quality reads (the ratio of the bases with quality value *Q* ≤ 5 greater than 50%) using the SOAPnuke software (http://soap.genomics.org.cn/) developed by BGIA. The remaining clean reads were mapped to maize B73 reference genome (RefGen_v3) and genes using BWA (version 0.7.10-r789) (Li & Durbin, 2009) and Bowtie2 (version 2.2.5) (Langmead & Salzberg, 2012), respectively.

### Gene expression analysis and identification of DEGs

The Bowtie2 aligner was used to map the clean reads on the genes and all the uniquely mapped reads were transformed into FPKM (fragments per kilobase of exon model per million mapped reads) using RSEM tool to estimate transcript expression levels in all samples (Li & Dewey, 2011). DESeq2 package (version 1.14.1) was applied to detect DEGs between each chosen sample pairs (Love, Huber & Anders, 2014). The results of all statistical tests were corrected for multiple testing using the Benjamini–Hochberg False Discovery Rate (FDR ≤ 0.001) (Benjamini & Yekutieli, 2001) and |Log_2_FC| ≥ 1 (FC = fold change).

### GO annotation and KEGG enrichment pathway analysis

Gene Ontology (GO) and functional enrichment analysis were conducted on all identified DEGs as described (Ashburner et al., 2000). Kyoto Encyclopedia of Genes and Genomes (KEGG) analysis utilizes hypergeometric test to search DEGs that are significantly enriched in certain KEGG pathways compared with other genes in the genome. GO::TermFinder (Module Version: 0.86, http://search.cpan.org/dist/GO-TermFinder/lib/GO/TermFinder.pm) was used for accessing GO information and finding signicicantly enriched GO terms (Boyle et al., 2004). KOBAS 2.0 (Xie et al., 2011) and KEGG database (http://www.genome.jp/kegg/genes.html) were used for KEGG analysis. GO categories with corrected *P* value ≤0.05 and KEGG pathways with *Q* value (*P* value after multiple hypothesis testing correction with a range between 0 and 1) ≤0.05 were defined as significantly enriched.

### qRT-PCR analysis

cDNA was synthesized using PrimeScript RT Master Mix Kit (Takara, Japan) following the protocols. Gene-specific primers were designed with Primer-BLAST (http://www.ncbi.nlm.nih.gov/tools/primer-blast/). The primer sequence of DEGs and the control gene were listed in Table S1. qRT-PCR were carried out using an CFX96 Real-Time PCR Detection System (Bio-Rad, USA). The mixed solution of each qRT-PCR reaction contained 1.0μL of cDNA template, 1.0μL of 10μmol/L forward and reverse primers each, and 5μL of SYBR Premix Ex Taq II (Takara, Japan) in a final volume of 10μL. PCRs were implemented as follows: 95 °C for 2 min, followed by 40 cycles of 95 °C for 10 s, 60 °C for 30 s. qRT-PCR was performed with three replicates for each sample. Quantification cycle (Cq) was used for the determination of relative expresssion levels normalized to reference genes. 18S was used as a reference gene for qRT-PCR of transcriptional factors, and Actin was used as a reference gene for verification of RNA-seq results.

## Results

### Phenotypic characterization of fertile and sterile spikelets

Because C48-2 and N48-2 are a pair of isonuclear-alloplasmic lines, no phenotypic variation were founded between the two lines (Fig. S1A). No emergence of anthers and lacking mature pollen grain lead to the complete male sterility of C48-2 line (Fig. S1B). With the development of microspores, no obvious variations were detected between the microspores of C48-2 and N48-2 plants until the MS stage (Fig. S1C). It is consistent with the previous cytological study results, i.e., the premature microspore abortion and tapetum degradation at MS stage is one of the prominent characteristics of CMS-C in maize (Chen & Duan, 1988). Moraes, Bered & Kaltchuk-Santos (2008) suggested spikelet lengths are reliable parameters for predicting the specific microspore developmental stage of maize under the same planting condition. As shown in Fig. S1D, the microspores of the upper florets will be at the PS stages and MS stages when the spikelet lengths are in the 5.9 ∼ 7.2 mm range and 9.2 ∼ 10.6 mm range, respectively. For more accurate microspore development stages, the spikelets at the lengths of 6.0 ∼ 7.0 mm and 9.5 ∼ 10.5 mm were sampled and used for the RNA-seq.

### Production of ready-to-analyze gene expression data

With the removal of low quality reads, a total of 326.75  ±  6.11, 322.23  ±  1.85, 327.95  ±  6.94 and 323.83  ±  4.02 million clean reads and 408.43  ±  7.64, 402.79  ±  2.31, 409.94  ±  8.68 and 404.79  ±  503 MB nucleotides were acquired for C48-2 at the pollen mother cell stage (PS-C), N48-2 at the pollen mother cell stage (PS-N), C48-2 at the mononuclear stage (MS-C) and N48-2 at the mononuclear stage (MS-N), respectively. A high proportion of the clean reads, 67.45% from PS-C, 68.69% from PS-N, 69.04% from MS-C and 69.99% from MS-N, were mapped to the maize reference genome sequence, and 73.94%∼75.82% of the clean reads were assigned to known genes via BLAST analysis (Table 1). Pearson correlation coefficient analysis revealed high correlations between biological replicates, indicating that the sequencing data were reliable (Fig. S2).

**Table 1:** Summary of sequencing results.

Sample ID	PS-C	PS-N	MS-C	MS-N
Total Reads (×10^5^)	326.75 ± 6.11	322.23 ± 1.85	327.95 ± 6.94	323.83 ± 4.02
Total Base Paires (×10^7^)	408.43 ± 7.64	402.79 ± 2.31	409.94 ± 8.68	404.79 ± 5.03
Total Mapped Reads (×10^5^)	220.40 ± 5.72	221.34 ± 2.09	226.37 ± 2.22	226.63 ± 2.56
(mapping to reference genome)	(67.45%)	(68.69%)	(69.04%)	(69.99%)
Total Unmapped Reads (×10^5^)	106.34 ± 1.69	100.89 ± 0.93	101.58 ± 4.78	97.19 ± 1.75
(mapping to reference genome)	(32.55%)	(31.31%)	(30.96)	(30.01%)
Total Mapped Reads (×10^5^)	241.58 ± 9.30	240.83 ± 3.82	248.68 ± 7.03	244.34 ± 4.43
(mapping to reference gene)	(73.94%)	(74.74%)	(75.82%)	(75.45%)
Total Unmapped Reads (×10^5^)	85.17 ± 8.80	81.40 ± 2.15	79.27 ± 1.22	79.49 ± 2.24
(mapping to reference genes)	(26.06%)	(25.26%)	(24.18%)	(24.55%)

### Identification of DEGs

**Figure 1:**
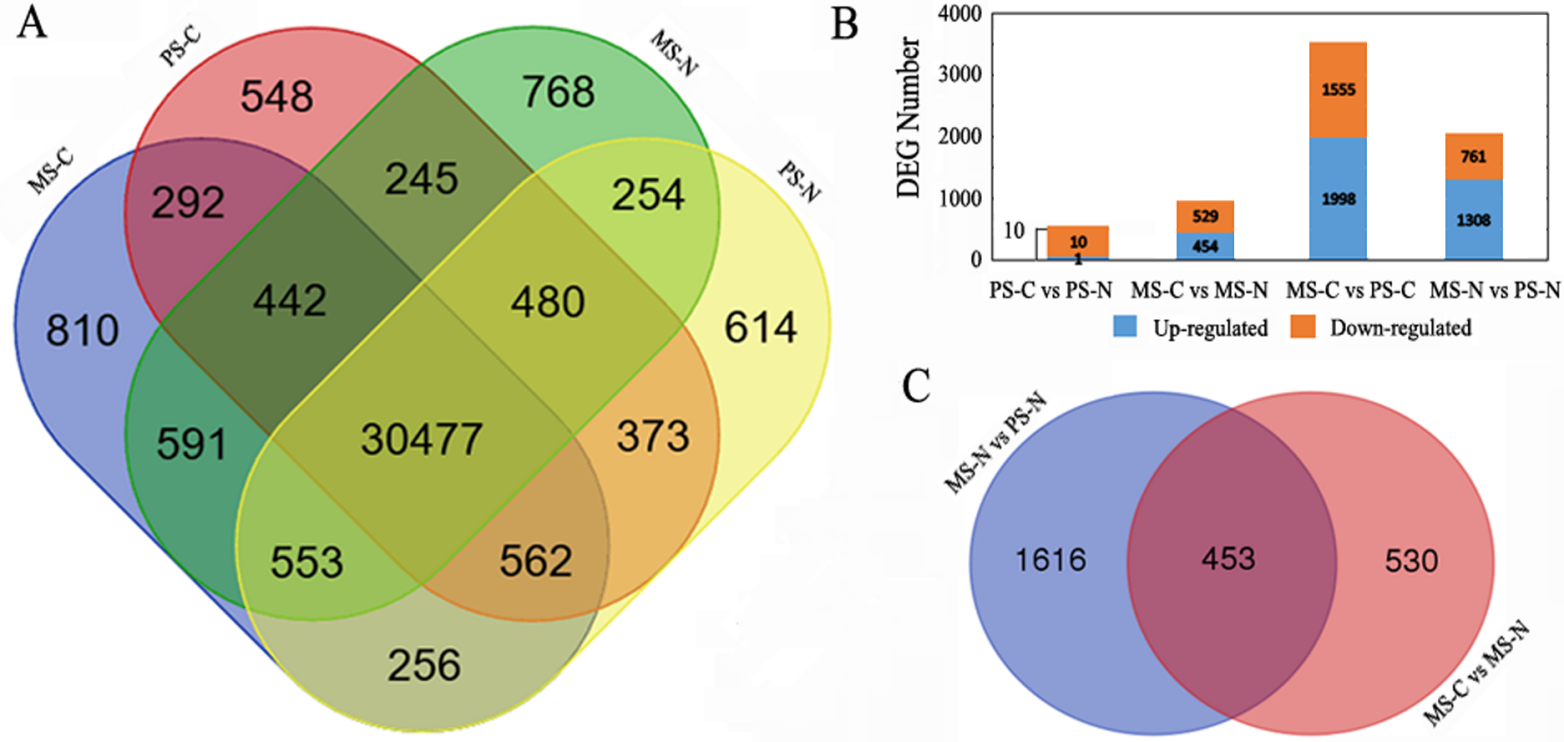
Statistical analysis of gene expression detected by RNA-seq. (A) Venn diagram of gene counts expressed at PS and MS stages for C48-2 and N48-2; (B) Number of total DEGs and down- or up- regulated DEGs, respectively; (C) Venn diagram displaying the relationship of DEGs between the two comparisons “MS-N vs PS-N” and “MS-C vs MS-N”. MS-C, PS-C, MS-N and PS-N represent the mononuclear stage of C48-2, pollen mother cell stage of C48-2, mononuclear stage of N48-2 and pollen mother cell stage of N48-2, respectively.

Gene expression of PS-C, PS-N, MS-C and MS-N were quantified and compared (Fig. 1A). At PS stage, 31,892 genes were detected in the two lines, 1,527 genes and 1,677 were uniquely expressed in C48-2 and N48-2, respectively. At MS stage, there were 1,920 genes specifically expressed in C48-2 and 1,747 in N48-2, and 32,063 genes were common expressed in the two lines. For the comparison between the two stages of the same line, C48-2 had 1,646 genes and 2,210 genes uniquely expressed at PS stage and MS stage, respectively, and 31,773 genes were commonly expressed at the two stages. As to N48-2, 1,805 genes were only expressed at PS stage and the number is 2,046 at MS stage, while 31,764 genes were found to be expressed at the two stages.

FDR ≤ 0.001 and ∣Log_2_FC∣ ≥ 1 were used as the threshold to screen the DEGs between the two lines at the same stages or between the different stages of the same lines. Compared with N48-2, 983 DEGs including 454 up-regulated and 529 down-regulated were identified at MS stage of C48-2, and only 11 DEGs, 1 up-regulated and 10 down-regulated, were found in C48-2 at PS stage (Fig. 1B). A large number of DEGs that is 3,553 and 2,069 showed significant expression difference between the two stages of the C48-2 line and N48-2 line, respectively (Fig. 1B).

### qRT-PCR verification on RNA-seq results and DEGs identification

**Figure 2:**
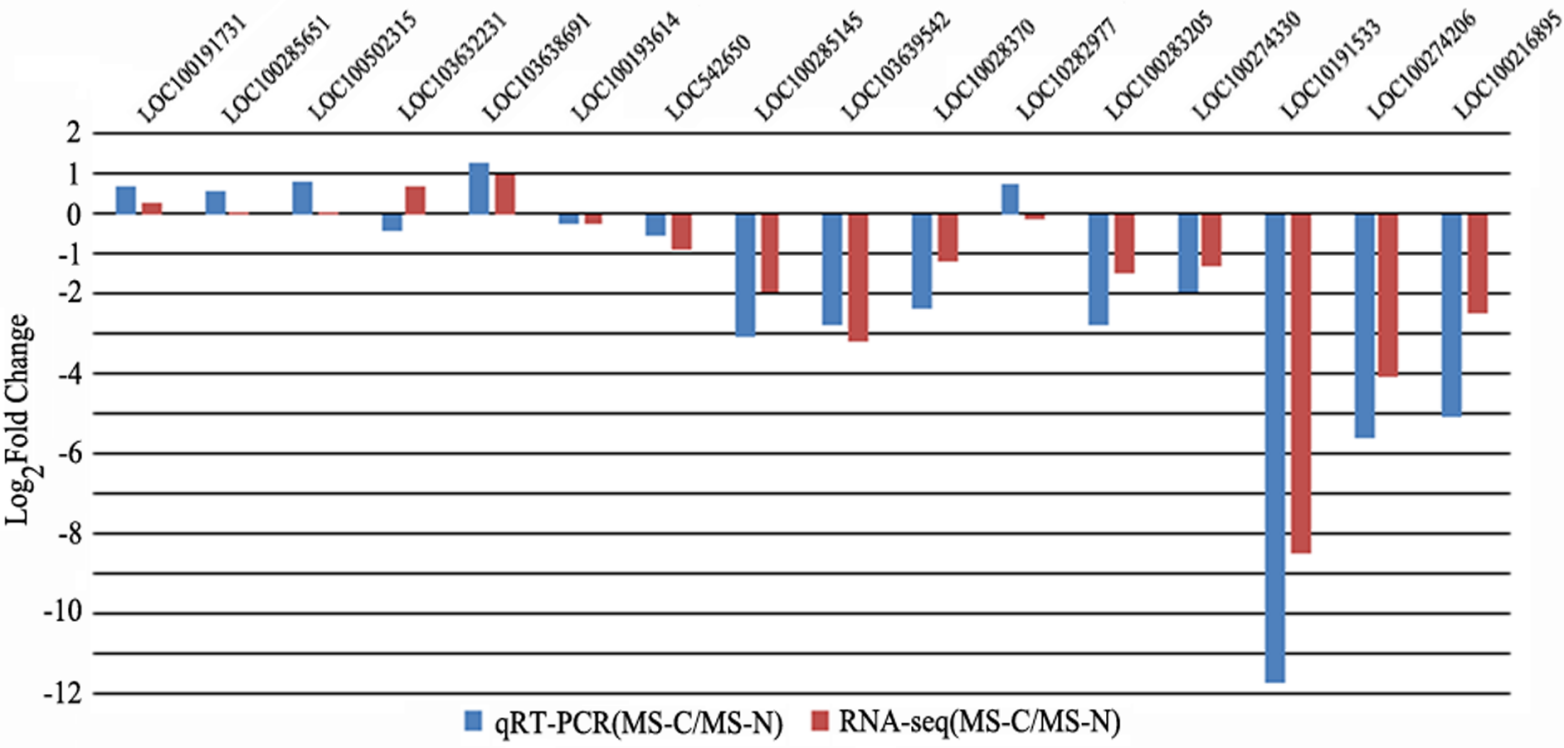
qRT-PCR verification on RNA-seq results and DEGs identification. *x*-axis represents genes ID, *y*-axis represents the logarithm of fold change; the blue column and red column respectively represents the qRT-PCR results and RNA-seq results of relative expression levels fold change between the two lines; |Log2Fold Change| ≥ 1 represents the gene is differentially expressed between the two lines.

qRT-PCR of 16 genes in C48-2 and N48-2 at the MS stage were applied to verify the filtering of DEGs using the same samples that used in RNA-seq. The qRT-PCR expression patterns of 14 genes were consistent with the RNA-seq results, and the coincidence rate was 87.50% (Fig. 2). As to the accuracy of filtering of DEGs, all the 9 genes except LOC103638691 displayed as DEGs by qRT-PCR were identified by RNA-seq analyses, and the coincidence rate was 88.89% (Fig. 2). These results indicated the RNA-seq data in the present study were reliable.

### DEGs expression patterns between different comparisons

Further, the normalized reads number of DEGs between C48-2 and N48-2 were 57.30  ±  89.82 for PS-C, 161.44  ±  197.18 for PS-N, 194.85  ±  817.49 for MS-C and 608.83  ±  3136.55 for MS-N (Table 2). The mean values for N48-2 were 2.82 and 3.12 times larger than that for C48-2 at PS stage and MS stage, respectively. It suggested that more gene expressions were down-regulated along with the substitution of C cytotype for N cytotype. Meanwhile, the normalized read numbers of DEGs between PS stage and MS stage were 161.09  ±  534.04 for PS-N, 495.37  ±  2458.30 for MS-N, 155.20  ±  406.47 for PS-C and 229.25  ±  940.67 for MS-C (Table 2). The mean value of normalized read numbers of DEGs in MS stage was 3.08 times larger than that in PS stage in N48-2. But the mean value was only increased about 48% from PS stage to MS stage in C48-2. This suggested that, along with spikelet development, more genes need to be activated in N48-2 and the activation of these genes expression was inhibited in C48-2. Collectively, in combination with the large standard deviations of the normalized read numbers, the results not only suggest reasonable coverage for the DEGs, but it’s also a reasonable indication that the inhibition of gene expression by C cytotype causes the microspore abortive at the whole genome level.

**Table 2:** Normalized reads number of DEGs between different comparisons.

Groups	Normalized reads number of DEGs			
	PS-C	PS-N	MS-C	MS-N
PS-C vs PS-N	57.30 ± 89.82	161.44 ± 197.18	/	/
MS-C vs MS-N	/	/	194.85 ± 817.49	608.83 ± 3136.55
PS-C vs MS-C	155.20 ± 406.47	/	229.25 ± 940.67	/
PS-N vs MS-N	/	161.09 ± 534.04	/	495.37 ± 2458.30

### Key DEGs selection

Because the C48-2 and N48-2 were isonuclear-alloplasmic lines, only 11 DEGs were detected between the two lines at PS stage, but the number of DEGs increased greatly to 983 at MS stage (Fig. 1B). The number of differentially expressed genes was consistent with the observed phenotypic differences between N cytotype and C cytotype, anther development at the early and late developmental stages. These results demonstrate the important role that mitochondrial retrograde regulation (MRR) at MS stage plays in the maize anther development, and also made it possible to draw the conclusion that there are no obvious changes at PS stage between the transcriptome levels of C48-2 and N48-2, and focusing the DEGs at MS stage is more reasonable.

In N48-2 line, 2,069 DEGs between PS stage and MS stage were detected (Fig. 1B). It suggests that the 2,069 DEGs should be related to the spikelet normal developments from PS to MS stage. Among the 2,069 DEGs, 453 DEGs were found to be significantly differentially expressed between C48-2 and N48-2 at the MS stage (Fig. 1C and Table S2). The 453 genes were thought to be the key DEGs that participated in the process or the causes of microspore abortion. Among the 453 DEGs, 385 (84.99%) genes were down-regulated and only 68 (15.01%) genes were up-regulated in C48-2 at MS stage (Table S2). It is worth noting that 92 (23.90%) of the 385 down-regulated genes and only 1 (1.47%) of the 68 up-regulated genes were differentially expressed more than ten times (Table S2). The utilization of the 453 DEGs for the following analyses will provide a narrow range for the identification of potential pollen sterility related genes and/or metabolic pathways.

### GO and KEGG enrichment analyses of the 453 DEGs

GO and KEGG analysis are widely used in the description of gene product functions (Ashburner et al., 2000; Kanehisa et al., 2004). As shown in Fig. S3, the GO enrichment of the 453 DEGs was analyzed. Six GO categories, i.e., “polyamine metabolic process” of biological process (BP), “Cytoplasmic part”, “Cytoplasm”, “Cytoplasmic vesicle” and “Vesicle” of cellular component (CC) and “Aspartate oxidase activity” of molecular function (MF) were significantly annotated (Table 3). The 453 DEGs were enriched in 78 pathways by KEGG analysis and the top 20 pathways enrichment was stated in Fig. S4. Seven pathways including “Nitrogen metabolism”, “Alanine, aspartate and glutamate metabolism”, “Fatty acid elongation”, “Plant-pathogen interaction”, “Cutin, suberine and wax biosynthesis”, “Biosynthesis of unsaturated fatty acids” and “Arginine and proline metabolism” were significantly enriched (Table 4).

**Table 3:** Significant terms of GO analysis of the 453 DEGs.

Accession	Description	DEGs Number	DEGs (log_2_(fold change))	DEGs (log_2_(fold change))	Corrected *P*-value
GO:0006595	Polyamine metabolic process	5		103638134 (−1.95), 100279222 (−1.49), 103633099 (−4.09), 100501649 (−1.96), 100501307 (−4.54)	2.14E−03
GO:0044444/ GO:0005737	Cytoplasmic part/Cytoplasm	75	103630336 (+1.18), 103647886 (+2.23), 100274575 (+1.01), 100272878 (+1.14), 100502492 (+1.28), 100194192 (+1.02), 103652814 (+3.43), 100284795 (+1.00), 100282067 (+1.38), 100191778 (+1.28), 542525 (+1.85), 100191593 (+1.85), 100285871 (+1.17), 100283978 (+1.32), 103644166 (+1.16), 100282311 (+1.08), 542671 (+1.69), 100273345 (+1.56), 542252 (+1.29), 100282076 (+1.55),	100280212 (−1.11), 100274597 (−1.61), 100502513 (−2.80), 103641988 (−4.04), 100501125 (−4.13), 100383497 (−1.96), 100277154 (−2.31), 100273256 (−3.53), 103630103 (−3.74), 100384494 (−3.65), 100191623 (−6.94), 100282491 (−6.29), 100282997 (−2.55), 103627899 (−2.09), 541796 (−2.22), 100284041 (−1.69), 100285183 (−2.48), 100382027 (−2.43), 103626422 (−2.23), 100285881 (−4.19), 100191895 (−1.21), 103639025 (−1.18), 100273983 (−3.55), 100281998 (−2.59), 100216656 (−1.51), 100283790 (−1.10), 103639307 (−1.39), 100501502 (−1.36), 100283569 (−2.89), 103642123 (−4.13), 542761 (−5.77), 100191963 (−3.33), 100276550 (−1.61), 100279948 (−1.74), 100383090 (−4.68), 100383115 (−6.00), 100283366 (−3.69), 100273547 (−2.07), 103626181 (−1.72), 542314 (−1.67), 103646055 (−6.47), 100284356 (−1.76), 541892 (−1.25), 100286025 (−7.15), 100382211 (−1.87), 100283909 (−1.49), 100272793 (−2.00), 100279917 (−1.22), 100283568 (−1.50), 103626492 (−1.27), 100192014 (−2.69), 100282493 (−6.09), 100280292 (−3.14), 100282284 (−3.37), 100284872 (−3.58)	4.20E−04/ 4.50E−04
GO:0031410/ GO:0031982	Cytoplasmic vesicle/Vesicle	64	103630336 (+1.18), 103647886 (+2.23), 100274575 (+1.01), 100272878 (+1.14), 100502492 (+1.28), 103644166 (+1.16), 100282311 (+1.08), 542671 (+1.69), 100194192 (+1.02), 103652814 (+3.43), 100273345 (+1.56), 100284795 (+1.00), 100282067 (+1.38), 100191778 (+1.28), 100282076 (+1.55), 542525 (+1.85), 100191593 (+1.85), 100285871 (+1.17), 100283978 (+1.32),	100280212 (−1.11), 100502513 (−2.80), 103639307 (−1.39), 103641988 (−4.04), 100501502 (−1.36), 103642123 (−4.13), 100283569 (−2.89), 100191963 (−3.33), 100501125 (−4.13), 100383497 (−1.96), 100277154 (−2.31), 100273256 (−3.53), 100276550 (−1.61), 100279948 (−1.74), 100383090 (−4.68), 103630103 (−3.74), 100384494 (−3.65), 100191623 (−6.94), 100383115 (−6.00), 100283366 (−3.69), 100282491 (−6.29), 100282997 (−2.55), 100273547 (−2.07), 103627899 (−2.09), 103626181 (−1.72), 100285183 (−2.48), 100382027 (−2.43), 542314 (−1.67), 103646055 (−6.47), 100284356 (−1.76), 541892 (−1.25), 100286025 (−7.15), 100285881 (−4.19), 100382211 (−1.87), 100272793 (−2.00), 100283568 (−1.50), 100191895 (−1.21), 103639025 (−1.18), 103626492 (−1.27), 100282493 (−6.09), 100192014 (−2.69), 100282284 (−3.37), 100280292 (−3.14), 100273983 (−3.55), 100281998 (−2.59)	6.80E−04
GO:0015922	Aspartate oxidase activity	2		103644300 (−4.44), 100279867 (−4.25)	1.95−02

**Notes.**

“+” means up-regulated in C48-2 and “−” means down-regulated in C48-2.

**Table 4:** Significant pathway enrichments of KEGG analysis of the 453 DEGs.

Pathway ID	Description	DEGs number	DEGs (log2(fold change))	DEGs (log2(fold change))	*Q* value
ko00910	Nitrogen metabolism	8	100285064 (+1.08), 100192349 (+1.33), 100192351 (+1.37)	100272754 (−2.50), 100501546 (−5.31), 100279972 (−5.32), 100274597 (−1.61), 542220 (−1.10)	2.22E−03
ko00250	Alanine, aspartate and glutamate metabolism	8	100192349 (+1.33), 100192351 (+1.37)	100037816 (−1.62), 100279867 (−4.25), 542220 (−1.10), 100216685 (−3.30), 732739 (−3.94), 103644300 (−4.44)	3.00E−03
ko00062	Fatty acid elongation	5		100286157 (−1.43), 100191179 (−2.10), 100274548 (−2.12), 542359 (−3.09), 103653730 (−2.92)	9.85E−03
ko04626	Plant-pathogen interaction	30	100279991 (+1.16), 542671 (+1.69), 100285871 (+1.17), 103626943 (+2.33), 103651407 (+1.87), 103633426 (+1.01),	100275623 (−1.50), 100384222 (−1.40), 103651071 (−2.59), 103630103 (−3.74), 100283091 (−1.90), 103649813 (−1.19), 100501502 (−1.36), 100282922 (−2.30), 103651392 (−1.83), 103632695 (−2.41), 100191429 (−1.36), 100273547 (−2.07), 103627039 (−2.02), 100274330 (−1.31), 103628553 (−2.03), 103626248 (−1.77), 100280377 (−1.23), 103640356 (−3.18), 103641871 (−1.38), 103654972 (−2.01), 100281107 (−2.01), 103639404 (−2.08), 103654474 (−3.57), 103626181 (−1.72)	1.60E−02
ko00073	Cutin, suberine and wax biosynthesis	6		100192014 (−2.69), 100283366 (−3.37), 103639542 (−3.21), 100501544 (−3.29), 100191290 (−1.50), 100280292 (−3.14)	2.87E−02
ko01040	Biosynthesis of unsaturated fatty acids	5		100191179 (−2.10), 100274548 (−2.13), 103640085 (−5.87), 542359 (−3.09), 103653730 (−2.92)	2.90E−02
ko00330	Arginine and proline metabolism	7		100501307 (−4.54), 103633099 (−4.09), 100501649 (−1.96), 103638134 (−1.95), 100279222 (−1.49), 100277253 (−1.24), 542220 (−1.10)	2.90E−02

**Notes.**

“+” means up-regulated in C48-2 and “−” means down-regulated in C48-2.

### The analyses of DEGs encoding transcription factors

As shown in Table 5, 18 of the 453 DEGs were identified as transcription factor genes. The expression levels of all the 18 transcription factors have no significant difference between C48-2 and N48-2 at PS stage, but all of them were down-regulated in C48-2 at MS stage except LOC100279991 and LOC100383937. The mRNA amount of five transcription factors, including four genes LOC100272446, LOC100216895, LOC100191533 and LOC100274206 encoding MYB protein, and one gene LOC732739 encoding LBD protein in C48-2 were estimated to be only 0.28% ∼17.63% of that in N48-2 at MS stage (Table 5).

**Table 5:** Statistics of the transcription factors identified from the 453 DEGs.

GeneID	TF family	Normalized reads number		log_2_(MS-C/MS-N)	Padj value	Differential expression model	*P* value	
		MS-C	MS-N					
100283422	Alfin-like	71.43	156.25	−1.13	9.83E−18	down		1.59E−06
100193098	AP2-EREBP	25.79	103.90	−2.01	3.26E−06	down		3.95E−08
100857063	ARF	158.09	676.77	−2.10	1.45E−26	down		2.07E−29
100274330	bHLH	2268.98	5639.82	−1.31	1.21E−11	down		6.27E−14
100282922	bHLH	410.12	2025.57	−2.30	6.15E−03	down		1.87E−04
100279991	bHLH	114.68	51.24	+1.16	1.82E−04	up		3.18E−06
100191923	C3H	304.23	925.61	−1.61	4.93E−08	down		4.21E−10
100275160	G2-like	16.60	39.38	−1.25	2.6E−02	down		1.12E−03
732739	LOB	75.37	1154.62	−3.94	4.74E−18	down		1.30E−20
100275160	MYB	16.60	39.38	−1.25	2.59E−02	down		1.12E−03
100272446	MYB	2.72	769.96	−8.15	1.70E−70	down		2.44E−74
100216895	MYB	30.21	171.37	−2.50	3.73E−03	down		1.02E−04
100191533	MYB	6.07	2141.97	−8.46	1.31E−64	down		2.99E−68
100274206	MYB	4.26	70.74	−4.05	4.87E−13	down		2.20E−15
100383937	NAC	60.89	29.73	+1.03	1.31E−02	up		4.68E−04
103626248	Tify	8.37	28.60	−1.77	5.24E−03	down		1.54E−04
100384222	Tify	175.71	463.25	−1.40	3.46E−04	down		6.43E−06
100275623	WRKY	56.54	159.40	−1.50	3.33E−05	down		4.87E−07

**Notes.**

MS-C means C48-2 at the MS stage, MS-N means N48-2 at the MS stage.

**Figure 3:**
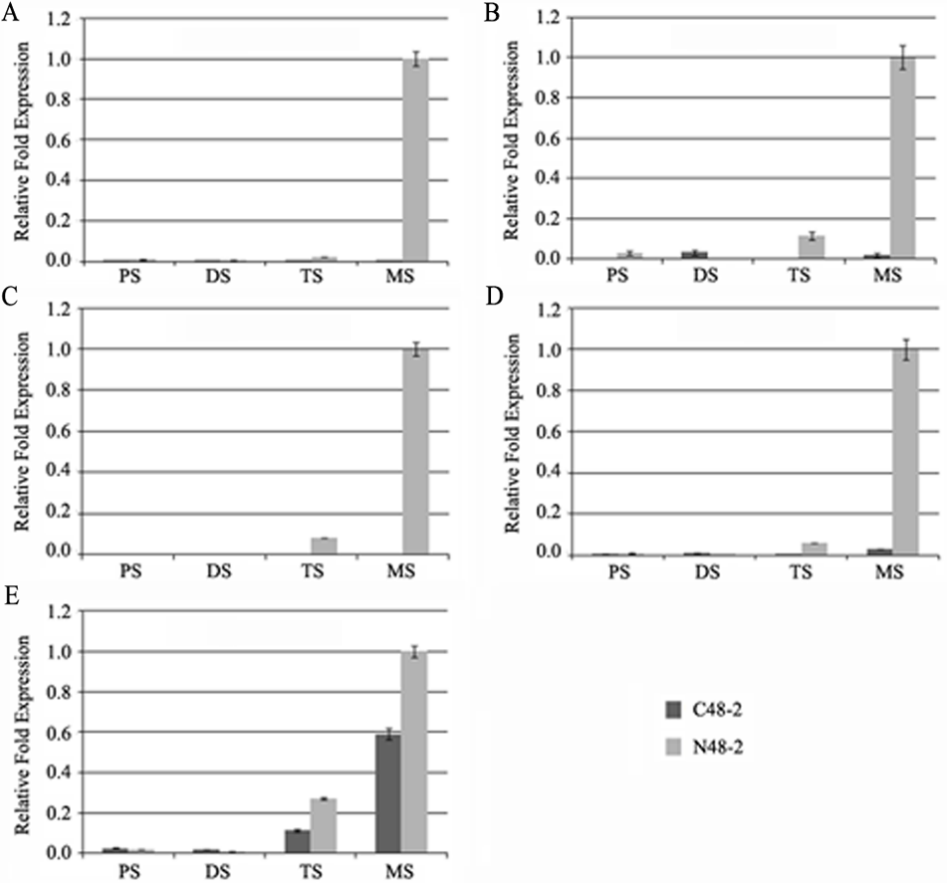
Comparative analyses of the five transcriptional factors expression levels between C48-2 and N48-2 along with the microspore development. (A) The expression level of LOC100191533; (B) The expression level of LOC100274206; (C) The expression level of LOC100272446; (D) The expression level of LOC100216895; (E) The expression level of LOC732739. PS, DS, TS and MS represents pollen mother cell stage, dyad stage, tetrad stage and mononuclear stage, respectively.

**Figure 4:**
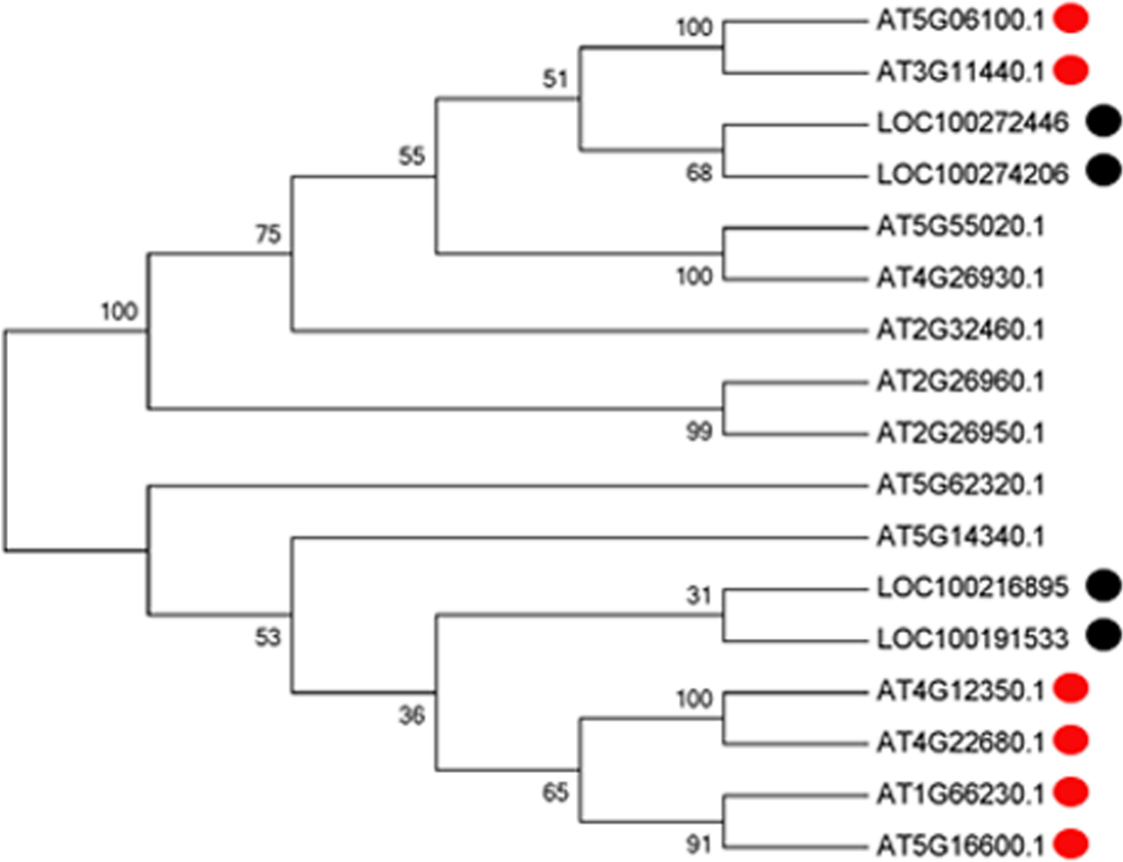
Phylogenetic analysis of the four MYB transcription factors with the 13 MYBs of Arabidopsis. Multiple sequence alignments of proteins were generated using ClustalW (version 7.2.5), and the phylogenetic tree was constructed with the neighbor-joining algorithm in MEGA (version 5.1). Bootstrap analysis was carried out with 1,000 replicates.

With the development of microspores in N48-2, the five genes displayed similar expression patterns, i.e., extreme low or even no expression at PS and DS stages then increased at the TS stage and reached a much higher level at the MS stage, but the expression levels were significantly lower than that in N48-2 at both TS and MS stages of C48-2, especially for the expression of the four MYB genes that were inhibited extremely at the two stages (Fig. 3). Phylogenetic analysis shows that LOC100272446 and LOC100274206 were homologous to MYB33 (at5g06100) and MYB65 (at3g11440); LOC100216895 and LOC100191533 were homologous to MYB42 (at4g12350), MYB85 (at4g22680), MYB20 (at1g66230) and MYB43 (at5g16600) (Fig. 4).

## Discussion

### Different stages of microspore development have different MRR performance in the CMS line

CMS traits were generated by the changes in mitochondrial function due to the rearrangement of the mitochondrial genome (Hu et al., 2014). The changes trigger off the alterations of the nuclear gene expression through MRR (Rhoads & Subbaiah, 2007; Fujii, Komatsu & Toriyama, 2007). In the present study, the comparative transcriptome analysis between the CMS-C line C48-2 and its isonuclear-alloplasmic maintainer line N48-2 were analyzed. No obvious variations were detected between the microspore of C48-2 and N48-2 plants until the MS stage (Fig. S1C). Meanwhile, the differential expression analyses only detected 11 DEGs at PS stage and the number increased greatly to 983 at the MS stage between the isonuclear-alloplasmic lines (Fig. 1B). As we know, the heritable alterations of phenotype result from the changes of gene expression. The huge difference of DEG numbers between the PS and MS stages not only indicated the rationality and reliability of the RNA-seq data, but also showed the space–time specificity of the MRR in maize CMS-C.

### Differentially expressed patterns of most DEGs that involved in the causes or process of microspore abortion were characterized as down-regulation

The cross-comparison of the DEGs from the two comparisons of “MS-N vs PS-N” and “MS-C vs MS-N” detected 453 DEGs that provided a narrow range for the identification of potential pollen sterility related genes and/or metabolic pathways (Fig. 1C). At MS stage, 84.99% of the 453 DEGs were down-regulated in C48-2 line (Table S2). Among the 453 DEGs, 16 out of 18 genes characterized as transcription factor genes were down-regulated in C48-2 at MS stage (Table 5). GO and KEGG enrichment analyses of the 453 DEGs found that the DEGs involved in “Polyamine metabolic process”, “Aspartate oxidase activity”, “Fatty acid elongation”, “Cutin, suberine and wax biosynthesis”, “Biosynthesis of unsaturated fatty acids” and “Arginine and proline metabolism” were all significantly down-regulated in C48-2 at the MS stage (Tables 3 and 4).

### The significantly down-regulated expression of MYB was essential for male sterility

The alteration of transcription factors expression often leads to the abnormal pollen development and the male sterility during plant growth. Millar & Gubler (2005) found that the *myb33 myb65* had a premeiotic abortion of pollen development because the tapetum undergoes hypertrophy at the pollen mother cell stage in Arabidopsis. A battery of SND1-regulated transcription factors, including MYB20 (at1g66230), MYB43 (at5g16600), MYB42 (at4g12350) and MYB85 (at4g22680), is required for normal secondary cell wall biosynthesis and secondary cell wall thickening of the endothecium is necessary for the release of pollen grains (Zhong et al., 2008; Keijzer, 1987; Millar & Gubler, 2005; Dawson et al., 1999; Steiner-Lange et al., 2003). In the present study, four genes encoding MYB transcription factors were homologous to Arabidopsis MYB33, MYB65, MYB42, MYB85, MYB20 and MYB43 and found to be absolutely suppressed or significantly down-regulated in C48-2 along with the microspore development (Figs. 3 and 4). We suggest that the dramatic decreases of these MYB mRNA amount at the TS and MS stages might be involved in the male sterility of C48-2 line.

### Down-regulation of DEGs involved in energy metabolism potentially related to CMS in maize

High respiration rate and great energy demand are usually observed during microspore development (Tadege & Kuhlemeier, 1997). ATP, the energy currency for the Cell, in male sterile lines was significantly decreased (Bergman et al., 2000; Teixeira, Knorpp & Glimelius, 2005; Li et al., 2015). NAD is essential for energy metabolism and electron transfer, de novo biosynthesis of NAD starts with the oxidation of L-Asp to α-iminosuccinate and catalyzed by L-Asp oxidase (AO) in plants (Katoh et al., 2006). In this study, two DEGs, LOC103644300 and LOC100279867, encoding AO protein were found involved in “Aspartate oxidase activity” and the mRNA amount of LOC103644300 and LOC100279867 in C48-2 were estimated to be only 4.60% and 5.24% of that in N48-2 (Table 3). We speculated that the suppressed expression of the genes that related to the energy supply might result in a shortage in energy required for microspore development and ultimately led to the male sterility of C48-2.

### Down-regulation of DEGs involved in polyamine metabolic pathway might resulted in the accumulation of ROS in C48-2 line

For the male fertility of higher plant, the importance of polyamines, i.e., putrescine, spermidine and spermine, has been widely demonstrated (Falasca et al., 2010; Aloisi et al., 2016; Song & Tachibana, 2007). In plants, the ornithine decarboxylase (ODC), arginine decarboxylase (ADC) and spermidine synthase (SPDS) were essential for the synthesis of putrescine (Aloisi et al., 2016). ADC and ODC enzymes were expressed at the mononuclear stage of Nicotiana tabacum microspore and inhibited activity of SPDS led to abnormal pollen grains in kiwifruit (Bokvaj, Hafidh & Honys, 2015; Falasca et al., 2010). 5 DEGs (LOC100501307 and LOC103633099 encoding ODC protein, LOC100501649 and LOC103638134 encoding ADC protein, and LOC100279222 encoding SPDS protein) that significantly enriched in “polyamine metabolic process” were all down-regulated in C48-2 at MS stage (Table 3). Particularly, the mRNA amount of LOC100501307 and LOC103633099 in C48-2 were estimated to be only 4.30% and 5.87% of that in N48-2 (Table 3). Therefore, we hypothesized these genes’ significant down-regulation in C48-2 might lead to the reduction of cellular polyamine levels. Polyamines can protect cellular constituents from reactive oxygen species (ROS) because of the powerful properties for hydroxyl radical scavenging and singlet oxygen quenching (Das & Misra, 2004). ROS induce programmed cell death (PCD) and high levels of ROS indiscriminately attack cellular constituents then leads to male sterility (Xie et al., 2014; Song et al., 2016). Our previous study found that more ROS were produced in C48-2 than that in N48-2 at MS stage (Huang et al., 2012). Collectively, it is reasonable to draw a conclusion that the sharp down-regulated expression of the polyamine metabolism related genes might result in the reduction of cellular polyamine levels and the balance between ROS production and scavenging were disturbed, then the higher concentrations of ROS were accumulated in C48-2 line at MS stage, eventually led to the male sterility.

### Down-regulation of DEGs involved in fatty acid synthesis might disrupt the pollen wall development

In most cases, the male sterility traits were on account of the defective pollen wall (Wu et al., 2015). The pollen wall consists of the exine and the intine (Dobritsa et al., 2011). The exine was derived from the tapetum and consists of sporopollenin (Jiang, Zhang & Cao, 2013). Sporopollenin biosynthesis is closely associated with fatty acid metabolism for wax and cutin formation (Morant et al., 2007; Dobritsa et al., 2009; Li et al., 2010). Here, six DEGs were significantly enriched in the fatty acid metabolism related “biosynthesis of unsaturated fatty acids” and “fatty acid elongation” (Table 4). The synthesized C16 and C18 fatty acids are transported to the endoplasmic reticulum and lengthened into very long chain fatty acids (VLCFAs) that are used for sporopollenin and/or wax biosynthesis (Jiang, Zhang & Cao, 2013). In the process of fatty acid elongation, the four enzymatic reactions catalyzed by β-ketoacyl-CoA synthase (KCS), β-ketoacyl-CoA reductases (KCR), β-hydroxyacyl -CoA dehydrase and trans-2-enoyl-CoA reductases (ECR) are the core biosynthetic activities (Costaglioli et al., 2005). In this study, LOC100286157 that encoding KCS protein, LOC103653730 (*GL8B*) and LOC542359 (*GL8*) that encoding KCR protein, LOC100274548 (*PAS2*) that encoding β-hydroxyacyl -CoA dehydrase, and LOC100191179 that encoding ECR protein were all down-regulated in C48-2 at MS stage (Table 4). In maize, studies had demonstrated GL8 and PAS2 are two essential enzymes in VLCFAs synthesis (Xu et al., 1997; Xu et al., 2002; Bach et al., 2008). Meanwhile, all the DEGs significantly enriched in “Cutin, suberine and wax biosynthesis” were down-regulated in C48-2 at MS stage (Table 4). Based on these results, it is also reasonable to draw another conclusion that the down-regulation of the genes involved in the fatty acid synthesis might disrupt the pollen wall development and then contribute to the pollen abortion.

### Analysis of DEGs involved in proline metabolism of pollen development

In a number of plant species, massive proline accumulation in anthers and pollen had been reported by different authors (Mattioli et al., 2012; Biancucci et al., 2015). It is not very clear, to date, the reason for such a massive proline accumulation in pollen. In Arabidopsis, aberrant and infertile pollen grains were found in the proline-deficient mutant p5cs1 p5cs2/P5CS2 that defective in proline biosynthetic enzymes, and exogenous proline supplied can partially complement both morphological and functional defects of pollen (Mattioli et al., 2012; Funck et al., 2012). This supports the idea that proline is required for pollen development at the genetic and molecular level. In maize, Zou et al. (2009) found that along with the maize microspore development, the proline content was gradually increase in the maintainer line, but the proline contents were significant reduced in the CMS-C line. In this study, seven DEGs were significantly enriched in “arginine and proline metabolism” pathway and all were down-regulated in C48-2 line compared with that of N48-2 line (Table 4). These results showed that the down-regulation of these genes expression might be correlated with the proline content reduction and then made the defects in pollen development and male fertility in C48-2 line.

## Conclusion

In the present study, the RNA-seq and comparative transcript profiling analyses of CMS-C line C48-2 and its isonuclear-alloplasmic maintainer line N48-2 were applied to investigate the maize CMS-C sterility associated genes and/or pathways. Finally, we found the four genes encoding MYB transcription factor, and the DEGs involved in energy metabolism related “Aspartate oxidase activity”, regulation of ROS concentration related “Polyamine metabolic process”, pollen wall development related “Cutin, suberine and wax biosynthesis”, “Fatty acid elongation” and “Biosynthesis of unsaturated fatty acids”, and pollen development related “Proline metabolism” might be the important factors contributing to the sterile trait of maize CMS-C. Although there is no direct evidence indicating that these transcription factors and metabolism pathways are responsible for the maize CMS-C, the related research results in other plant species strongly suggest such a role. This study might shed some light on how the CMS generated and maintained, and provided some important and critical foothold for the molecular mechanism study of the male sterility of maize CMS-C. Based on the results of the present study, further precise analyses about the molecular mechanism of how these genes and metabolic pathways lead to the male sterility of CMS-C are needed.

##  Supplemental Information

10.7717/peerj.3408/supp-1 Figure S1 Phenotypic characterization of N48-2 and C48-2 lines. (A) Phenotypes of plants at 75 days after sow; (B) Phenotypes of fertility; (C) Phenotypes of microspore at different developmental stages; (D) Phenotype of spikelets. PS, DS, TS and MS represents pollen mother cell stage, dyad stage, tetrad stage and mononuclear stage, respectively.

10.7717/peerj.3408/supp-2 Figure S2 Pearson correlation coefficient analysis of the transcriptome data for the three biological replicates. MS-C, PS-C, MS-N and PS-N represent the mononuclear stage of C48-2, pollen mother cell stage of C48-2, mononuclear stage of N48-2 and pollen mother cell stage of N48-2, respectively.

10.7717/peerj.3408/supp-3 Figure S3 GO analysis results of the 453 DEGs. The x-axis corresponds to the top GO terms, and the y-axis shows the number of DEGs. BP, biological process; CC, cellular component and MF, molecular function.

10.7717/peerj.3408/supp-4 Figure S4 KEGG analysis results of the 453 DEGs. The y-axis corresponds to the KEGG pathway, and the x-axis shows the rich factor. The color of the dot represents the *Q* value, and the size of the dot represents the number of DEGs mapped to the reference pathways.

10.7717/peerj.3408/supp-5 Table S1 Summary of primers used in qRT-PCR.

10.7717/peerj.3408/supp-6 Table S2 Staticstics of the 453 DEGs.

10.7717/peerj.3408/supp-7 Supplemental Information 1 Identified DEGs between different comparisons Identified DEGs between different comparisons. DESeq2 package (version 1.14.1) was applied to detect DEGs between each chosen sample pairs. The results of all statistical tests were corrected for multiple testing using the Benjamini-Hochberg False Discovery Rate (FDR ≤ 0.001) and |*Log*_2_*FC*| ≥ 1 (FC = fold change).

## Additional Information and Declarations

### Data Availability

Paired-end RNA-seq raw data of 3 biological replicates of MS-C: MS-C_1_1 (http://dx.doi.org/10.6084/m9.figshare.4905758); MS-C_1_2 (http://dx.doi.org/10.6084/m9.figshare.4907921); MS-C_2_1 (http://dx.doi.org/10.6084/m9.figshare.4906679); MS-C_2_2 (http://dx.doi.org/10.6084/m9.figshare.4907939); MS-C_3_1 (http://dx.doi.org/10.6084/m9.figshare.4909289); MS-C_3_2 (http://dx.doi.org/10.6084/m9.figshare.4909292). Paired-end RNA-seq raw data of 3 biological replicates of PS-C: PS-C_1_1 (http://dx.doi.org/10.6084/m9.figshare.4909301); PS-C_1_2 (http://dx.doi.org/10.6084/m9.figshare.4909505); PS-C_2_1 (http://dx.doi.org/10.6084/m9.figshare.4909304); PS-C_2_2 (http://dx.doi.org/10.6084/m9.figshare.4909307); PS-C_3_1 (http://dx.doi.org/10.6084/m9.figshare.4909331); PS-C_3_2 (http://dx.doi.org/10.6084/m9.figshare.4909337). Paired-end RNA-seq raw data of 3 biological replicate of MS-N: MS-N_1_1 (http://dx.doi.org/10.6084/m9.figshare.4906874); MS-N_1_2 (http://dx.doi.org/10.6084/m9.figshare.4906871); MS-N_2_1 (http://dx.doi.org/10.6084/m9.figshare.4906964); MS-N_2_2 (http://dx.doi.org/10.6084/m9.figshare.4906961); MS-N_3_1 (http://dx.doi.org/10.6084/m9.figshare.4906994); MS-N_3_2 (http://dx.doi.org/10.6084/m9.figshare.4907006). Paired-end RNA-seq raw data of 3 biological replicate of PS-N: PS-N_1_1 (http://dx.doi.org/10.6084/m9.figshare.4907165); PS-N_1_2 (http://dx.doi.org/10.6084/m9.figshare.4907165); PS-N_2_1 (http://dx.doi.org/10.6084/m9.figshare.4907165); PS-N_2_2 (http://dx.doi.org/10.6084/m9.figshare.4907174); PS-N_3_1 (http://dx.doi.org/10.6084/m9.figshare.4907345); PS-N_3_2 (http://dx.doi.org/10.6084/m9.figshare.4907348).
