# Supplementary figures and images for: Comparative transcriptome analysis of isonuclear-alloplasmic lines unmask key transcription factor genes and metabolic pathways involved in sterility of maize CMS-C
Source: PeerJ. 2017 May 30;5:e3408. doi: 10.7717/peerj.3408 (PMC5452966; doi:10.7717/peerj.3408)

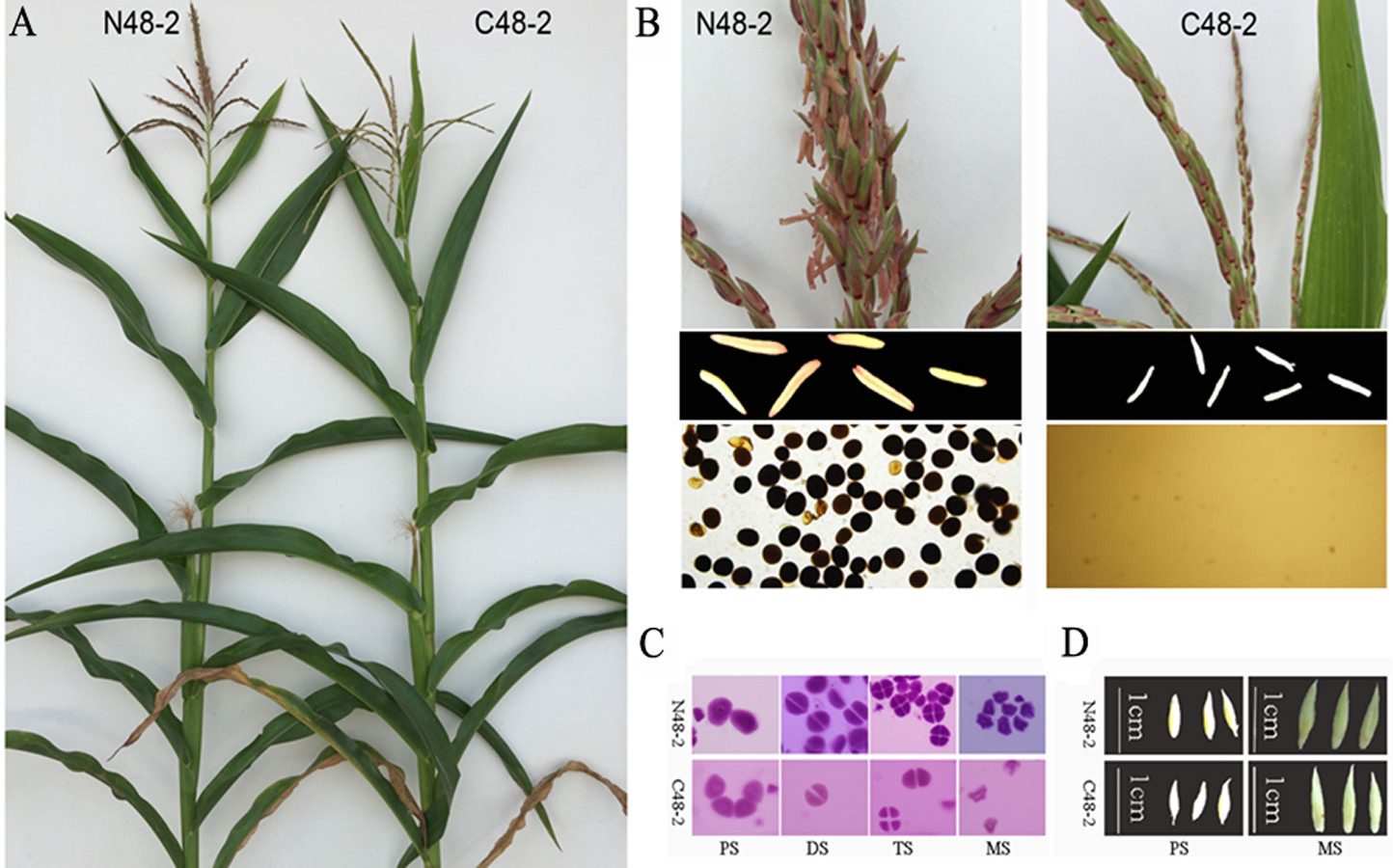

Supplement: Figure S1 — (A) Phenotypes of plants at 75 days after sow; (B) Phenotypes of fertility; (C) Phenotypes of microspore at different developmental stages; (D) Phenotype of spikelets. PS, DS, TS and MS represents pollen mother cell stage, dyad stage, tetrad stage and mononuclear stage, respectively. [file peerj-05-3408-s001.png]

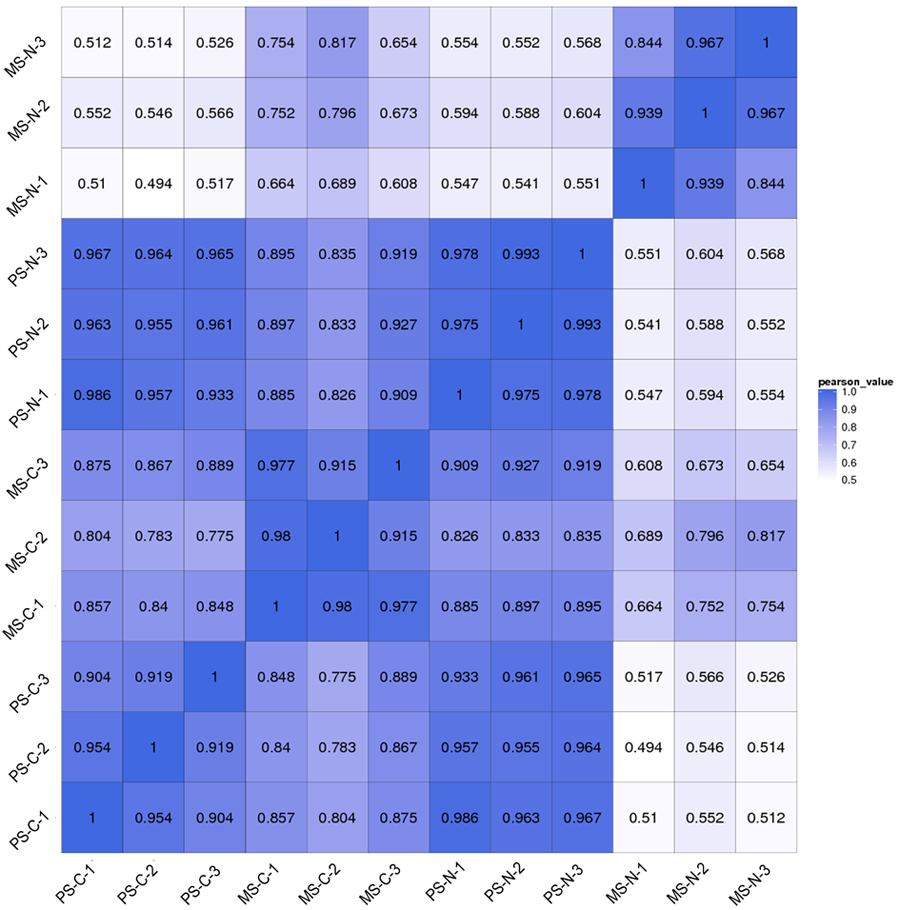

Supplement: Figure S2 — MS-C, PS-C, MS-N and PS-N represent the mononuclear stage of C48-2, pollen mother cell stage of C48-2, mononuclear stage of N48-2 and pollen mother cell stage of N48-2, respectively. [file peerj-05-3408-s002.png]

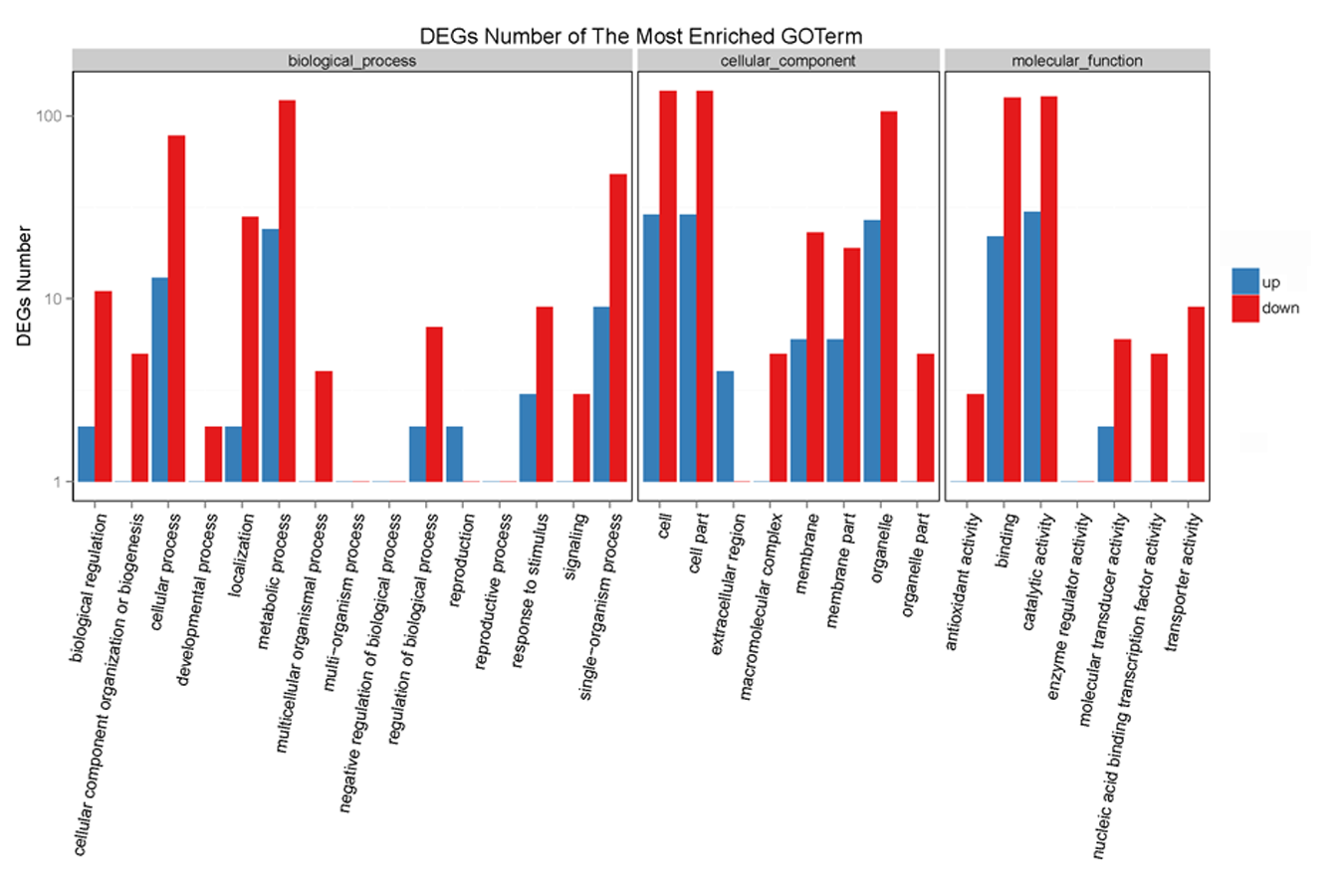

Supplement: Figure S3 — The x-axis corresponds to the top GO terms, and the y-axis shows the number of DEGs. BP, biological process; CC, cellular component and MF, molecular function. [file peerj-05-3408-s003.png]

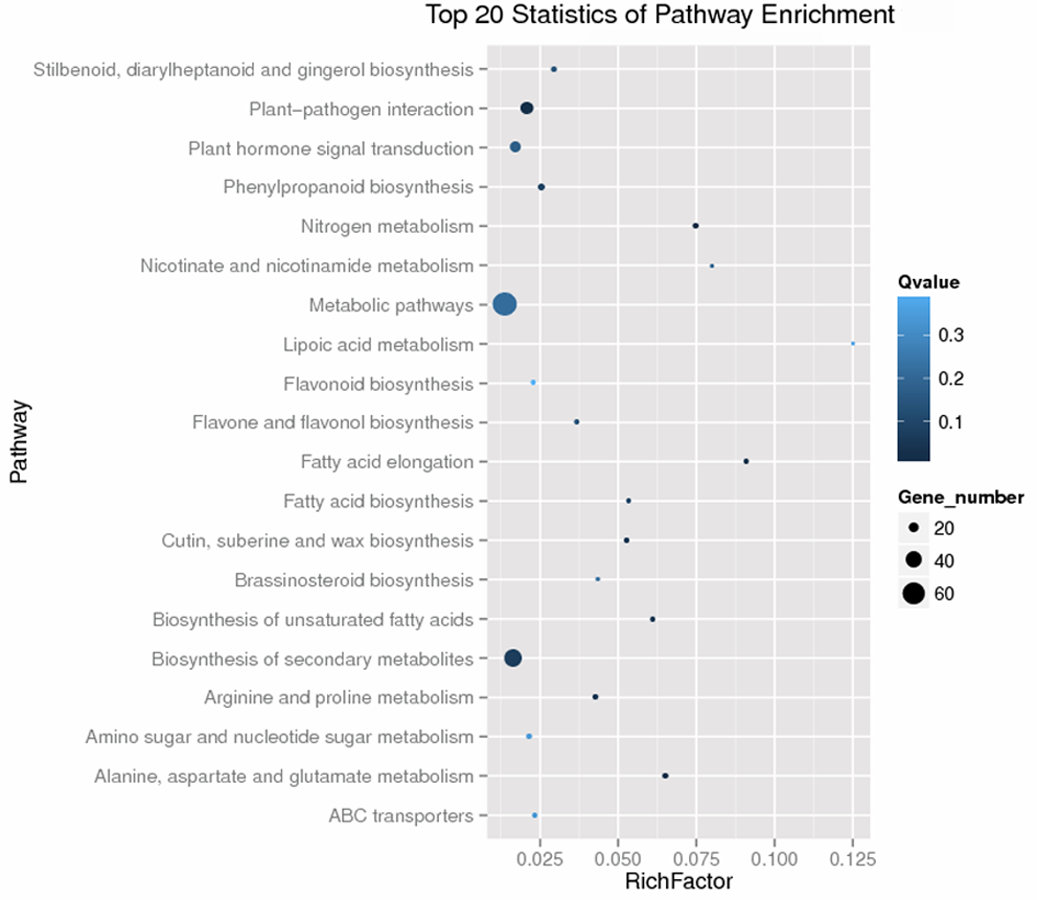

Supplement: Figure S4 — The y-axis corresponds to the KEGG pathway, and the x-axis shows the rich factor. The color of the dot represents the Q value, and the size of the dot represents the number of DEGs mapped to the reference pathways. [file peerj-05-3408-s004.png]
