# Supplementary material for: Comparative transcriptome analysis of isonuclear-alloplasmic lines unmask key transcription factor genes and metabolic pathways involved in sterility of maize CMS-C
Source: PeerJ. 2017 May 30;5:e3408. doi: 10.7717/peerj.3408 (PMC5452966; doi:10.7717/peerj.3408)
Supplement: Table S1 [file peerj-05-3408-s005.docx]

**Table S1. Summary of primers used in qRT-PCR**

| Gene ID | Forward Primer (5’-3’) | Reverse Primer (5’-3’) |
| --- | --- | --- |
| 100191731 | TGCTGCGTCACCTGATATCT | GCTGCCGACTCATGTTTAGG |
| 100285651 | TCTTTCCGCTCCCCAAGATT | GGATGAATGGGCTGTGTTCC |
| 100502315 | TGCCTCCAGATGCACCTAAA | GCTGGCTGTGTTGTTGATCA |
| 103632231 | GCAGGGTATGGAATGCAAGG | TGTCGAAAAGCTCTTCCCCT |
| 103638691 | TGTCACATGGCAAGAAACGG | CTACCAATAGATGCACGGCG |
| 100193614 | GTTGCTGCAGTCCCATATGG | ATGGTTTGTGCATTGGTCCC |
| 542650 | TCGATTGCAAATCTACCCCC | AGCATCGGCAGGAACATTCT |
| 100285145 | TCACGCCCTTGTGATCATCC | TGCAGGCATTTCCCATGAATA |
| 103639542 | ACGAAACATCGTGGCTGTCT | CCGGAGTACCATTTGCACCT |
| 100283370 | CTACAGCTACGCCTACGACG | TTAGTTCGCAGGGCAGAAGG |
| 100282977 | AGAAGAACTCACTTTCCACAGTT | GCTGCCTCCTCATCCAACTT |
| 100283205 | GTGAACAAGATGCTGCCCAAG | AGATGTAGAGGACCTGTGGCG |
| 100274330 | CTGGAGGTCACCAACGTCAA | AGCGAGTCCCTCAGTCTGTC |
| 100304121 | CTCAGGTCGACGCTTCTAGG | TGCAAGTATCCACACACAGC |
| 732739 | GCTGTCCAGGAGCTATGACG | CCAGACTTCAAGAACGGGCT |
| 100216895 | GGCTTCGACCAAGAAGACGA | GAAAACATGGAGGCTACGGC |
| 100282922 | GGGGAATCAATTTGGCCAGC | CGGATCAGCTACTACGCCTG |
| 100274206 | CATGCCGTGTGTGAAGCATT | AGGTTCACGCTAATCTGCCA |
| 100191533 | GACGATTTTCTCGACCACGA | GTCGTCTAGTAGCCCCATCT |
| 100272446 | CCTCCACCGTGAATAGGACG | GAAAACGCATTGGTTTCCCGA |
| 18S | CTGAGAAACGGCTACCACA | CCCAAGGTCCAACTACGAG |
| Actin | CTGAGGTTCTATTCCAGCCATCC | GCCACCACTGAGGACAACATTAC |
