# Supplementary material for: Comparative transcriptome analysis of isonuclear-alloplasmic lines unmask key transcription factor genes and metabolic pathways involved in sterility of maize CMS-C
Source: PeerJ. 2017 May 30;5:e3408. doi: 10.7717/peerj.3408 (PMC5452966; doi:10.7717/peerj.3408)
Supplement: Table S2 [file peerj-05-3408-s006.pdf]

Table S2. Statistics of the 453 DEGs

GeneID	Relative expression level		Log_2(MS-C/MS-N)	Padj	Differential expresion model	P value
	MS-C	MS-N				
103652814	36.63	3.40	3.43	2.96059E-09	Up	2.13199E-11
103636474	85.42	10.14	3.07	7.955E-15	Up	3.00069E-17
100275282	165.80	22.08	2.91	1.04642E-17	Up	2.96037E-20
100276638	928.93	165.88	2.49	0.002415825	Up	6.10963E-05
103635063	58.94	11.33	2.38	5.86629E-07	Up	6.08523E-09
103626943	24.39	4.83	2.34	0.001537877	Up	3.59486E-05
103646792	181.68	36.41	2.32	1.23128E-07	Up	1.11538E-09
100280082	26.84	5.40	2.31	0.000255537	Up	4.59315E-06
103647886	5.99	1.27	2.23	0.014384873	Up	0.000524521
100286852	20.93	5.21	2.01	0.009224497	Up	0.000301297
100277644	13.01	3.29	1.98	0.013289989	Up	0.000477382
100275312	10.96	2.80	1.97	0.029119372	Up	0.00129645
100381631	199.34	52.62	1.92	2.36229E-07	Up	2.23951E-09
103651407	17.13	4.70	1.87	0.011687565	Up	0.000406129
542525	35.53	9.82	1.85	0.000997556	Up	2.13799E-05
100191593	16.56	4.58	1.85	0.022644149	Up	0.000945394
100282577	62.82	17.74	1.82	0.000852673	Up	1.77556E-05
542671	17.21	5.32	1.69	0.02400853	Up	0.001010589
103653588	16.94	5.25	1.69	0.043584469	Up	0.002213225
100274322	57.74	18.25	1.66	0.000271008	Up	4.93318E-06
100285804	83.58	27.03	1.63	1.4991E-06	Up	1.68784E-08
100282243	178.00	59.15	1.59	2.66321E-07	Up	2.54951E-09
100273345	23.88	8.10	1.56	0.018213257	Up	0.000712521
100282076	229.12	78.34	1.55	2.36229E-07	Up	2.24118E-09
100272880	114.05	39.20	1.54	0.000105834	Up	1.72993E-06
103647433	203.74	71.90	1.50	1.27379E-08	Up	9.79168E-11
100276273	183.24	64.98	1.50	8.398E-08	Up	7.4155E-10
100283620	111.77	39.87	1.49	2.76289E-07	Up	2.66073E-09
103646319	430.58	155.19	1.47	1.72664E-08	Up	1.37661E-10
100278708	56.21	20.29	1.47	0.002772904	Up	7.17117E-05
103647908	83.91	31.98	1.39	0.008608539	Up	0.000277734
100281437	41.32	15.82	1.39	0.027947812	Up	0.001229116
103633355	62.78	24.10	1.38	0.00448819	Up	0.000127743
100282067	178.87	68.68	1.38	1.16226E-05	Up	1.55438E-07
100192351	99.95	38.76	1.37	0.000130672	Up	2.18446E-06
100194314	30.47	11.90	1.36	0.029977862	Up	0.001352859
100279427	122.54	47.99	1.35	0.002280336	Up	5.70833E-05
100283208	3798.37	1496.95	1.34	7.00155E-08	Up	6.1024E-10
100192349	860.63	341.48	1.33	1.91008E-09	Up	1.33729E-11
100283978	183.43	73.33	1.32	0.007440729	Up	0.000233041
100216857	55.59	22.30	1.32	0.011860871	Up	0.000414185
100502492	197.91	81.04	1.29	1.02177E-05	Up	1.3548E-07
100191778	113.87	46.81	1.28	2.0075E-05	Up	2.7995E-07
103629798	37.56	15.47	1.28	0.034596245	Up	0.00164212
103638141	78.93	32.72	1.27	0.015468146	Up	0.000577724
103653067	86.88	36.23	1.26	0.00121393	Up	2.67804E-05
542252	317.26	132.61	1.26	7.10903E-08	Up	6.21639E-10
103630336	92.10	40.50	1.19	0.038482564	Up	0.001892567

103650987	32.65	14.44	1.18	0.039851788	Up	0.001981655
100285871	179.87	80.19	1.17	0.00517106	Up	0.000152055
103644166	81.84	36.55	1.16	0.019420643	Up	0.000776959
100279991	114.68	51.24	1.16	0.000182496	Up	3.17597E-06
100279695	52.90	23.70	1.16	0.018490652	Up	0.000726543
100285138	197.49	88.92	1.15	1.17197E-05	Up	1.57741E-07
100501166	126.95	57.36	1.15	2.20678E-05	Up	3.09633E-07
100272878	75.36	34.29	1.14	0.001147197	Up	2.5046E-05
100282473	316.54	145.97	1.12	6.91396E-09	Up	5.19623E-11
100280121	125.95	58.49	1.11	0.000100906	Up	1.63693E-06
100282311	735.82	346.57	1.09	1.10673E-10	Up	6.57829E-13
100285064	906.62	430.14	1.08	0.00012137	Up	2.01161E-06
100194091	44.67	21.39	1.06	0.03519029	Up	0.001682834
103633426	392.48	188.75	1.06	6.52717E-06	Up	8.2816E-08
103637861	258.94	124.57	1.06	1.01076E-05	Up	1.33557E-07
100383937	60.89	29.73	1.03	0.013083521	Up	0.000468096
100194192	478.62	235.76	1.02	2.19586E-06	Up	2.57029E-08
100274575	101.40	50.47	1.01	0.014440655	Up	0.000527381
100383538	187.61	93.63	1.00	0.004041607	Up	0.00011226
100284795	1023.69	511.55	1.00	0.001257846	Up	2.79649E-05
100856910	259.83	522.35	-1.01	0.002246371	Down	5.57196E-05
103652932	45.51	93.12	-1.03	0.002247765	Down	5.58184E-05
103632651	37.03	75.83	-1.03	0.014170603	Down	0.000514684
100280875	80.74	165.81	-1.04	0.023060114	Down	0.000966055
100283585	1452.66	3000.85	-1.05	3.45134E-19	Down	8.99394E-22
100384255	69.23	143.05	-1.05	0.004572964	Down	0.000130809
100304404	498.75	1042.42	-1.06	0.000107395	Down	1.75851E-06
100283790	176.32	369.93	-1.07	5.57475E-07	Down	5.75094E-09
103629543	204.07	431.04	-1.08	0.001168743	Down	2.56166E-05
100281466	318.46	679.75	-1.09	2.59026E-15	Down	9.17851E-18
542220	499.33	1070.99	-1.10	2.61741E-08	Down	2.14665E-10
100283406	55.74	119.85	-1.10	0.000122191	Down	2.03221E-06
103644012	32.87	70.70	-1.11	0.014513252	Down	0.000530861
100280212	137.72	296.61	-1.11	1.27924E-09	Down	8.84653E-12
103640855	217.28	468.30	-1.11	0.004380572	Down	0.000124047
100276266	172.23	371.87	-1.11	0.010319568	Down	0.000345617
103627764	337.15	728.89	-1.11	5.1574E-05	Down	7.88481E-07
100502532	121.01	261.65	-1.11	0.011083241	Down	0.000382279
100274678	158.99	344.34	-1.11	0.000152915	Down	2.60001E-06
100217290	45.96	99.63	-1.12	0.006100884	Down	0.000184278
100283422	71.43	156.25	-1.13	9.83442E-05	Down	1.58783E-06
103648097	312.51	688.33	-1.14	1.33879E-05	Down	1.82489E-07
100283424	475.70	1057.77	-1.15	0.000944709	Down	2.00043E-05
103633153	466.89	1043.69	-1.16	2.91363E-08	Down	2.42289E-10
103639025	27.13	61.34	-1.18	0.033657063	Down	0.001585039
100274509	132.19	299.08	-1.18	1.16916E-06	Down	1.27627E-08
103649813	51.60	117.74	-1.19	0.012924627	Down	0.000460196
100284770	278.48	637.64	-1.20	3.16679E-10	Down	1.99994E-12
100191895	85.21	197.49	-1.21	0.000338232	Down	6.2538E-06
100191302	217.86	505.14	-1.21	3.043E-07	Down	2.96526E-09
100501509	28.59	66.47	-1.22	0.002477482	Down	6.30096E-05
100283348	656.40	1529.20	-1.22	6.86811E-11	Down	3.94493E-13
100279917	379.77	885.50	-1.22	7.81777E-05	Down	1.23319E-06
103644989	14.52	33.91	-1.22	0.0289154	Down	0.001280759

100280377	24.17	56.57	-1.23	0.020054634	Down	0.000815504
100277891	109.69	256.95	-1.23	0.006960274	Down	0.000216203
100277253	741.20	1755.20	-1.24	3.13355E-06	Down	3.79672E-08
103626829	45.00	106.68	-1.25	2.90543E-05	Down	4.16793E-07
100275160	16.60	39.38	-1.25	0.025928421	Down	0.001117726
541892	1783.90	4234.08	-1.25	1.00113E-35	Down	8.29654E-39
100281758	31.34	74.49	-1.25	0.00049633	Down	9.63046E-06
100281686	238.46	566.97	-1.25	0.00116539	Down	2.55098E-05
103638712	65.44	155.74	-1.25	0.000135876	Down	2.2831E-06
103649729	155.79	373.20	-1.26	3.89608E-05	Down	5.71152E-07
103626492	69.86	168.02	-1.27	2.26826E-07	Down	2.13902E-09
100278237	97.50	234.79	-1.27	0.002032162	Down	4.9419E-05
100279842	17.25	41.57	-1.27	0.020606897	Down	0.000843261
100126914	160.54	387.25	-1.27	4.49911E-06	Down	5.55415E-08
100280406	66.84	161.29	-1.27	0.000177566	Down	3.06514E-06
103630789	31.92	77.37	-1.28	0.001683592	Down	3.98839E-05
100274363	215.16	521.88	-1.28	0.001919298	Down	4.61807E-05
100276564	428.13	1049.26	-1.29	6.11209E-21	Down	1.37982E-23
100280133	221.87	551.10	-1.31	2.82323E-05	Down	4.02581E-07
100279260	55.03	136.75	-1.31	0.002107061	Down	5.16731E-05
100274330	2268.97	5639.82	-1.31	1.21256E-11	Down	6.27174E-14
103644988	31.34	78.38	-1.32	6.19158E-05	Down	9.56507E-07
103647971	95.77	239.83	-1.32	0.002907063	Down	7.61781E-05
100273615	81.13	205.52	-1.34	0.003577549	Down	9.69171E-05
100382805	542.42	1381.63	-1.35	1.3276E-06	Down	1.4682E-08
100500995	15.35	39.33	-1.36	0.011017044	Down	0.000377162
100191429	645.87	1659.44	-1.36	0.011066574	Down	0.000380721
100501502	14.65	37.69	-1.36	0.030346451	Down	0.001379699
100382938	116.90	303.89	-1.38	0.000306511	Down	5.62326E-06
103650464	32.47	84.42	-1.38	0.002254342	Down	5.61753E-05
100275584	92.92	242.33	-1.38	0.002258098	Down	5.63976E-05
103641871	73.44	191.69	-1.38	1.18395E-05	Down	1.60241E-07
103647718	25.60	67.08	-1.39	0.010774783	Down	0.000365174
103635762	278.70	731.29	-1.39	4.02778E-10	Down	2.60124E-12
100191873	10.48	27.49	-1.39	0.024011354	Down	0.001011394
103639307	89.01	233.82	-1.39	2.93963E-07	Down	2.85613E-09
100193190	130.00	341.52	-1.39	1.93328E-11	Down	1.011E-13
100193686	144.87	381.35	-1.40	1.18395E-05	Down	1.60368E-07
100384222	175.71	463.25	-1.40	0.000345597	Down	6.42921E-06
100273243	547.50	1445.83	-1.40	1.96704E-07	Down	1.84372E-09
103638587	193.00	510.83	-1.40	2.92375E-05	Down	4.21093E-07
100193545	15.67	41.53	-1.41	0.017474369	Down	0.000675625
100192940	10.85	28.85	-1.41	0.020327103	Down	0.000828907
100384577	121.36	323.73	-1.42	1.84014E-06	Down	2.09811E-08
100280135	15.83	42.38	-1.42	0.019876425	Down	0.000805417
103641797	10.80	28.96	-1.42	0.041070945	Down	0.002062115
100286157	21.69	58.59	-1.43	0.002707992	Down	6.9646E-05
100274081	716.20	1941.29	-1.44	1.64287E-16	Down	5.07031E-19
100191808	45.01	122.12	-1.44	2.19586E-06	Down	2.57174E-08
103650442	28.27	76.85	-1.44	0.001016773	Down	2.18789E-05
100285001	96.81	264.73	-1.45	0.000218883	Down	3.87802E-06
100125643	28.04	76.73	-1.45	4.51475E-05	Down	6.74858E-07
103636483	23.69	65.14	-1.46	0.027588805	Down	0.001208513
100278599	14.47	39.84	-1.46	0.02945448	Down	0.001316733

100285416	617.13	1704.32	-1.47	4.17419E-12	Down	2.03974E-14
100278114	12.57	34.89	-1.47	0.010219256	Down	0.000341382
100273763	513.02	1424.06	-1.47	0.016138985	Down	0.000610158
100274815	34.20	95.04	-1.47	0.001627951	Down	3.81936E-05
100279222	154.11	431.45	-1.49	0.037054919	Down	0.001800119
103654228	40.63	113.94	-1.49	1.13344E-06	Down	1.23081E-08
100283909	130.68	367.17	-1.49	2.08056E-08	Down	1.68257E-10
100275623	56.54	159.40	-1.50	3.32839E-05	Down	4.84126E-07
100283205	19425.24	54902.15	-1.50	0.002252592	Down	5.60026E-05
100283568	126.94	358.97	-1.50	5.36915E-08	Down	4.61826E-10
100191290	37.21	105.51	-1.50	0.001951846	Down	4.73543E-05
100382699	31.29	88.77	-1.50	0.002841871	Down	7.38201E-05
103640212	9.54	27.09	-1.51	0.030139182	Down	0.001368543
103632953	26.97	76.66	-1.51	0.003297712	Down	8.76399E-05
100216656	76.03	217.04	-1.51	3.09736E-05	Down	4.48752E-07
100283628	49.93	143.40	-1.52	8.4422E-06	Down	1.09044E-07
100281682	212.96	612.03	-1.52	0.018154928	Down	0.00070972
103626591	45.50	132.67	-1.54	1.65888E-07	Down	1.53698E-09
100282962	89.17	266.19	-1.58	1.32853E-08	Down	1.02504E-10
103654812	274.46	827.13	-1.59	4.41154E-23	Down	7.81606E-26
100280331	87.63	265.07	-1.60	1.67634E-08	Down	1.33172E-10
103653111	55.25	167.30	-1.60	0.001310144	Down	2.9502E-05
100382783	13.31	40.32	-1.60	0.006539243	Down	0.000200509
103633417	38.91	118.12	-1.60	8.79093E-06	Down	1.14553E-07
100284471	30.93	93.92	-1.60	0.00091848	Down	1.93701E-05
100191923	304.23	925.61	-1.61	4.93234E-08	Down	4.21435E-10
100274597	17.85	54.41	-1.61	0.033266324	Down	0.001556274
100276550	50.63	154.44	-1.61	0.001854083	Down	4.43467E-05
103626306	6.49	19.87	-1.62	0.044863707	Down	0.002302322
100037816	27.86	85.45	-1.62	0.000515124	Down	1.00834E-05
100217237	79.02	245.53	-1.64	1.06504E-05	Down	1.41522E-07
542314	61.69	195.99	-1.67	6.7684E-10	Down	4.48725E-12
103654924	103.15	330.54	-1.68	6.14397E-06	Down	7.76028E-08
542246	5.13	16.45	-1.68	0.043237494	Down	0.002189428
100284041	91.43	294.97	-1.69	1.1769E-05	Down	1.5874E-07
103640897	7.46	24.08	-1.69	0.031389261	Down	0.00144236
103626181	19.63	64.61	-1.72	0.000103551	Down	1.6867E-06
100193178	185.59	614.33	-1.73	4.25643E-09	Down	3.10165E-11
103645841	54.80	183.27	-1.74	0.000445507	Down	8.51701E-06
100279948	2681.30	8995.99	-1.75	1.62148E-11	Down	8.43314E-14
100284356	9.34	31.58	-1.76	0.004159723	Down	0.000116136
103646269	23.53	79.70	-1.76	0.010809845	Down	0.000366671
103626248	8.37	28.60	-1.77	0.005241095	Down	0.000154264
100381544	6.11	20.96	-1.78	0.014340788	Down	0.000522504
100280210	24.19	83.88	-1.79	2.73585E-05	Down	3.89339E-07
100284225	6.96	24.15	-1.80	0.005938985	Down	0.000178879
103636839	16.56	57.54	-1.80	2.31314E-05	Down	3.25217E-07
103638848	7.56	26.38	-1.80	0.008781638	Down	0.000284726
100384311	11.13	39.12	-1.81	0.001882215	Down	4.51809E-05
100278059	9.77	34.48	-1.82	0.010875366	Down	0.00037138
542365	2943.70	10399.91	-1.82	3.01106E-25	Down	4.81852E-28
103651392	145.69	517.16	-1.83	3.20215E-10	Down	2.04058E-12
100280347	14.68	52.27	-1.83	0.002036413	Down	4.95806E-05
103639813	4.00	14.33	-1.84	0.048867408	Down	0.00254573

100501494	280.85	1008.10	-1.84	8.28494E-24	Down	1.42052E-26
100501908	96.12	346.53	-1.85	6.81087E-16	Down	2.31609E-18
103627817	96.55	348.35	-1.85	8.19373E-14	Down	3.3483E-16
103626550	9.96	36.19	-1.86	0.000746575	Down	1.53394E-05
100382211	7.02	25.62	-1.87	0.01547433	Down	0.000578422
103638086	8.85	32.90	-1.90	0.004898611	Down	0.000142364
100281771	3.26	12.17	-1.90	0.030457839	Down	0.001386504
100283091	187.67	702.04	-1.90	4.33796E-34	Down	4.09078E-37
100282936	134.39	503.21	-1.90	0.0011324	Down	2.46582E-05
100279849	12.50	47.30	-1.92	0.00372392	Down	0.00010184
100285935	22.34	84.88	-1.93	4.25301E-08	Down	3.62176E-10
103634688	4.08	15.57	-1.93	0.031812238	Down	0.001467703
100284722	16.06	61.29	-1.93	4.76285E-05	Down	7.18633E-07
100194100	4.10	15.82	-1.95	0.010592088	Down	0.000356561
100275115	31.10	120.31	-1.95	3.78935E-10	Down	2.43643E-12
103638134	1686.65	6536.89	-1.95	0.001358042	Down	3.08522E-05
100279833	4.20	16.33	-1.96	0.011877782	Down	0.000415115
103635831	8.19	31.84	-1.96	0.001330314	Down	3.01083E-05
100383497	52.31	203.53	-1.96	0.044671642	Down	0.002281788
100501649	961.80	3750.56	-1.96	0.001534647	Down	3.58292E-05
100381989	6.90	26.90	-1.96	0.006115296	Down	0.000184888
103651202	2.24	8.76	-1.97	0.034596245	Down	0.00164127
103654021	1.28	5.02	-1.98	0.041762539	Down	0.002101613
103636179	5.14	20.29	-1.98	0.004648916	Down	0.000133247
103647431	257.13	1016.73	-1.98	7.75806E-05	Down	1.21933E-06
100383632	216.62	857.69	-1.99	3.98051E-11	Down	2.22947E-13
103638032	1.61	6.38	-1.99	0.039857206	Down	0.001985229
100284273	22.22	88.53	-1.99	8.56726E-07	Down	9.13182E-09
100272793	5721.17	22823.55	-2.00	0.035954243	Down	0.001732264
100281107	56.71	227.77	-2.01	0.010596501	Down	0.000357613
100193098	25.79	103.90	-2.01	3.25538E-06	Down	3.95364E-08
103654972	73.34	295.81	-2.01	2.37383E-09	Down	1.69589E-11
103635615	2.07	8.35	-2.01	0.033906642	Down	0.001601637
103633134	4.45	18.00	-2.02	0.010715072	Down	0.000362232
103627039	10.34	41.96	-2.02	0.002102114	Down	5.14806E-05
100384270	128.54	522.40	-2.02	9.66461E-11	Down	5.68929E-13
103633084	25.28	102.85	-2.02	1.39239E-06	Down	1.54383E-08
100280206	76.07	309.82	-2.03	1.60673E-08	Down	1.26724E-10
100382108	313.23	1278.85	-2.03	2.14713E-11	Down	1.14738E-13
103628553	33.18	135.60	-2.03	2.65125E-05	Down	3.76541E-07
103633344	1.16	4.76	-2.03	0.033833128	Down	0.001594468
100502310	17.02	69.72	-2.03	2.72179E-06	Down	3.25893E-08
103633013	40.15	165.18	-2.04	2.67017E-08	Down	2.20518E-10
103647851	1.22	5.02	-2.04	0.032448906	Down	0.001505887
103640584	5.36	22.18	-2.05	0.004110837	Down	0.000114463
100127513	1.56	6.50	-2.06	0.030139182	Down	0.001367042
103648597	1.20	5.04	-2.07	0.02945448	Down	0.001317965
100273547	5.72	24.04	-2.07	0.002763188	Down	7.12235E-05
100216764	6.15	25.98	-2.08	0.005364773	Down	0.000158518
103639404	1.53	6.45	-2.08	0.027085735	Down	0.001180367
103638045	1.85	7.82	-2.08	0.022838013	Down	0.000955445
103627899	6.24	26.57	-2.09	0.005137564	Down	0.00015074
100381369	64.93	276.53	-2.09	0.00167012	Down	3.94693E-05
103632719	46.54	198.85	-2.10	0.024417179	Down	0.001038257

100857063	158.09	676.71	-2.10	1.45128E-26	Down	2.07362E-29
100191179	1587.09	6810.27	-2.10	0.000692105	Down	1.40423E-05
100502386	1.89	8.16	-2.11	0.020007904	Down	0.00081246
103627992	98.64	426.05	-2.11	1.33851E-17	Down	3.82498E-20
100382901	59.98	260.43	-2.12	3.83686E-07	Down	3.80462E-09
100274548	1193.63	5215.95	-2.13	1.85355E-35	Down	1.58903E-38
100284426	30.73	135.37	-2.14	2.90543E-05	Down	4.16254E-07
100276698	11.25	50.03	-2.15	2.09612E-06	Down	2.42593E-08
100384123	4.62	20.96	-2.18	0.003884003	Down	0.00010755
103649305	1.37	6.26	-2.19	0.01770274	Down	0.000685972
100277639	110.19	502.38	-2.19	2.14485E-10	Down	1.31778E-12
100282648	449.69	2069.70	-2.20	3.45134E-19	Down	9.07365E-22
100284326	16.62	76.50	-2.20	1.102E-09	Down	7.55786E-12
541796	803.10	3751.96	-2.22	6.41499E-26	Down	9.8991E-29
103626422	11.18	52.31	-2.23	0.00020437	Down	3.58586E-06
103640165	66.05	309.39	-2.23	2.61115E-16	Down	8.3571E-19
100274135	272.88	1284.71	-2.24	7.98812E-05	Down	1.26234E-06
103632322	1.94	9.16	-2.24	0.014207903	Down	0.000516444
103640604	42.58	204.08	-2.26	9.74572E-07	Down	1.04715E-08
100277514	39.38	188.78	-2.26	6.98768E-11	Down	4.03358E-13
100381397	90.71	436.13	-2.27	1.26695E-13	Down	5.24968E-16
103634448	15.10	72.62	-2.27	0.000177568	Down	3.07499E-06
103641486	3.73	18.23	-2.29	0.001951846	Down	4.72905E-05
100284244	11204.54	54719.46	-2.29	3.15109E-07	Down	3.07959E-09
103628094	42.09	207.40	-2.30	2.41998E-13	Down	1.04423E-15
103627046	2.82	13.90	-2.30	0.009312146	Down	0.000306023
100282922	410.12	2025.57	-2.30	0.00614717	Down	0.000186557
103641948	1.29	6.39	-2.30	0.010386627	Down	0.00034816
103650488	7.48	37.01	-2.31	0.000640945	Down	1.28394E-05
100277154	4543.55	22522.20	-2.31	0.008975083	Down	0.000292381
100285734	311.71	1549.91	-2.31	1.49205E-06	Down	1.67564E-08
103655639	21.96	109.80	-2.32	1.35825E-12	Down	6.32664E-15
103631929	1.56	7.89	-2.33	0.008956597	Down	0.000291523
103646489	26.84	135.98	-2.34	4.3132E-09	Down	3.16766E-11
100382074	144.37	736.48	-2.35	5.88011E-23	Down	1.0586E-25
100283024	144.13	735.94	-2.35	0.000475946	Down	9.16693E-06
100192720	41.54	215.26	-2.37	3.96306E-12	Down	1.92334E-14
103647062	1.34	7.02	-2.39	0.006792182	Down	0.0002104
100501255	16.77	88.34	-2.40	8.95529E-08	Down	7.93319E-10
103653109	19.06	101.02	-2.41	3.94682E-07	Down	3.97006E-09
103632695	82.03	436.90	-2.41	8.74414E-05	Down	1.39575E-06
103630380	1.90	10.19	-2.42	0.005773481	Down	0.000172574
100382027	1.76	9.47	-2.43	0.005478781	Down	0.000162513
103653694	13.82	75.05	-2.44	5.30133E-08	Down	4.54477E-10
103630813	19.24	104.64	-2.44	1.07424E-10	Down	6.35442E-13
100384495	52.41	285.45	-2.45	2.27473E-12	Down	1.07906E-14
100383488	598.54	3282.53	-2.46	0.004808629	Down	0.000139249
103632429	1.29	7.06	-2.46	0.004653857	Down	0.000133655
103641576	4.50	24.77	-2.46	4.60452E-05	Down	6.92112E-07
100191633	80.07	442.19	-2.47	0.000400622	Down	7.54443E-06
103646464	1888.50	10501.49	-2.48	1.43388E-20	Down	3.40093E-23
103644206	1.37	7.65	-2.48	0.004165891	Down	0.000116784
100285183	323.41	1806.12	-2.48	4.73567E-06	Down	5.9003E-08
100283944	1342.83	7594.24	-2.50	2.02291E-21	Down	4.2524E-24

100272754	467.70	2652.15	-2.50	4.66317E-06	Down	5.79665E-08
100216895	30.21	171.37	-2.50	0.003729073	Down	0.000102088
100384599	16.91	96.06	-2.51	1.44104E-05	Down	1.98074E-07
100216674	41.06	238.84	-2.54	3.35081E-17	Down	9.95839E-20
100282997	1120.95	6549.54	-2.55	0.001937197	Down	4.66752E-05
100283948	22.11	130.24	-2.56	4.3221E-16	Down	1.42036E-18
100282312	59.61	351.08	-2.56	0.00276787	Down	7.15024E-05
100273315	533.29	3162.40	-2.57	0.000181846	Down	3.15427E-06
100192694	95.72	572.16	-2.58	2.19247E-13	Down	9.33526E-16
100286341	3.21	19.17	-2.58	0.000852673	Down	1.7763E-05
100281998	23.10	138.86	-2.59	0.000529676	Down	1.03986E-05
103651071	47.50	286.10	-2.59	5.70017E-10	Down	3.71389E-12
103626380	3.97	24.14	-2.60	0.000389279	Down	7.28633E-06
103640901	23.53	143.91	-2.61	8.12058E-20	Down	2.0421E-22
100279722	89.25	553.48	-2.63	9.06475E-19	Down	2.40905E-21
103652002	113.46	705.24	-2.64	5.26037E-06	Down	6.59913E-08
103655673	2.58	16.08	-2.64	0.001704142	Down	4.04681E-05
103647839	87.02	555.17	-2.67	1.9999E-28	Down	2.51459E-31
100192502	19.08	122.06	-2.68	7.56939E-12	Down	3.80698E-14
103647067	1.46	9.37	-2.68	0.001246929	Down	2.76644E-05
100192014	241.31	1554.87	-2.69	1.11565E-45	Down	5.73861E-49
100276850	26.06	173.27	-2.73	9.89399E-22	Down	1.97914E-24
100502513	505.82	3511.29	-2.80	1.10719E-06	Down	1.19914E-08
100272346	8.27	57.47	-2.80	6.88141E-06	Down	8.77038E-08
103633275	20.64	151.22	-2.87	5.17911E-13	Down	2.3532E-15
100283569	9.46	70.19	-2.89	1.33601E-08	Down	1.03463E-10
100193580	154.12	1146.04	-2.89	3.0365E-27	Down	4.25183E-30
100192089	700.44	5212.58	-2.90	4.26514E-05	Down	6.30129E-07
103653730	187.43	1419.17	-2.92	1.59198E-06	Down	1.80152E-08
103656021	1.97	15.66	-2.99	8.49915E-05	Down	1.34795E-06
100383387	267.98	2214.85	-3.05	3.93812E-07	Down	3.95005E-09
542359	348.45	2965.40	-3.09	1.43565E-06	Down	1.6E-08
100280292	940.51	8283.94	-3.14	9.5492E-08	Down	8.51389E-10
103640356	8.39	75.88	-3.18	2.9974E-08	Down	2.50112E-10
103632480	12.90	116.69	-3.18	5.05533E-05	Down	7.65652E-07
100383994	4.73	42.78	-3.18	2.38076E-06	Down	2.81658E-08
100279802	12.46	114.98	-3.21	5.59222E-18	Down	1.55011E-20
103639542	2460.79	22776.61	-3.21	8.5009E-06	Down	1.10045E-07
103631726	5.94	55.44	-3.22	2.33023E-09	Down	1.65141E-11
100501544	1807.06	17656.28	-3.29	3.78827E-06	Down	4.64414E-08
100283735	230.86	2262.55	-3.29	5.82813E-09	Down	4.34687E-11
100216685	119.40	1178.53	-3.30	6.96698E-33	Down	6.76908E-36
103630156	22.54	224.35	-3.32	1.7066E-06	Down	1.93977E-08
100191963	206.71	2080.27	-3.33	1.39394E-05	Down	1.91201E-07
100384001	176.79	1780.24	-3.33	1.43133E-63	Down	4.09022E-67
100285344	50.35	516.40	-3.36	1.22975E-61	Down	3.8656E-65
100191756	111.78	1152.90	-3.37	3.5698E-41	Down	2.14225E-44
103639541	80.57	831.31	-3.37	4.90161E-26	Down	7.28364E-29
100283366	413.10	4265.18	-3.37	1.09332E-05	Down	1.45592E-07
100282284	1.94	20.14	-3.37	5.62993E-06	Down	7.09493E-08
103635020	11.27	117.90	-3.39	1.12625E-14	Down	4.31268E-17
100304091	17.69	185.51	-3.39	9.86216E-18	Down	2.76188E-20
103644321	1.89	20.05	-3.40	1.17272E-06	Down	1.28351E-08
542430	18.17	195.38	-3.43	9.29617E-14	Down	3.82536E-16

100191933	382.78	4188.58	-3.45	3.02541E-15	Down	1.10662E-17
100273383	53.20	594.30	-3.48	4.25994E-09	Down	3.11638E-11
100191699	21.09	239.56	-3.51	2.62207E-07	Down	2.50263E-09
103649544	3.20	36.91	-3.53	1.67634E-08	Down	1.32914E-10
100273256	252.03	2905.32	-3.53	3.70711E-07	Down	3.64419E-09
100273983	300.48	3516.60	-3.55	4.23798E-15	Down	1.58649E-17
100382213	52.67	620.58	-3.56	1.99192E-08	Down	1.60519E-10
103654474	12.27	145.56	-3.57	2.71863E-15	Down	9.78875E-18
100284872	10.04	120.04	-3.58	2.75058E-14	Down	1.0847E-16
100384494	2.16	27.07	-3.65	3.90914E-07	Down	3.90981E-09
100276545	24.51	311.60	-3.67	5.05175E-09	Down	3.73894E-11
100192059	91.42	1169.38	-3.68	1.31435E-10	Down	7.92503E-13
103630103	24.78	331.49	-3.74	3.85912E-07	Down	3.84876E-09
100501856	27.43	368.65	-3.75	7.25297E-11	Down	4.20744E-13
100276688	5.30	72.27	-3.77	1.53167E-13	Down	6.43413E-16
103638322	1.67	23.47	-3.82	3.55467E-08	Down	3.00675E-10
100383822	198.02	2832.41	-3.84	8.12748E-11	Down	4.7612E-13
103654037	1.92	27.58	-3.84	4.23955E-09	Down	3.07723E-11
103637588	3.29	47.83	-3.86	1.08771E-09	Down	7.39773E-12
100383186	4.00	60.36	-3.92	3.66568E-10	Down	2.34644E-12
732739	75.37	1154.62	-3.94	4.74253E-18	Down	1.30103E-20
103646079	2.25	34.44	-3.94	2.32423E-09	Down	1.64053E-11
100276718	33.91	533.00	-3.97	3.42552E-11	Down	1.89904E-13
100277380	34.60	548.76	-3.99	6.64982E-64	Down	1.71025E-67
103641988	2.68	43.89	-4.04	1.25452E-09	Down	8.63975E-12
100274544	16.32	269.68	-4.05	1.11567E-24	Down	1.81726E-27
103637524	3.53	58.49	-4.05	6.69565E-10	Down	4.41989E-12
100274206	4.26	70.74	-4.05	4.8722E-13	Down	2.19983E-15
100274857	7.24	121.14	-4.07	1.55425E-12	Down	7.28401E-15
100277429	9.64	164.03	-4.09	1.35573E-17	Down	3.91291E-20
103633099	14.84	252.89	-4.09	2.3387E-09	Down	1.6641E-11
100501125	10.16	177.66	-4.13	7.31598E-26	Down	1.14985E-28
103642123	5.66	99.22	-4.13	3.36525E-21	Down	7.37174E-24
100285881	22.09	403.88	-4.19	2.58987E-11	Down	1.40617E-13
100279867	108.92	2077.16	-4.25	1.62E-202	Down	4.6294E-207
100273778	12.90	255.67	-4.31	2.35552E-22	Down	4.57722E-25
103632851	2.29	45.51	-4.31	7.84429E-12	Down	3.99007E-14
103636283	1.70	34.21	-4.33	2.58987E-11	Down	1.40171E-13
103629791	1.89	40.00	-4.40	1.18369E-11	Down	6.0886E-14
103654996	17.22	367.26	-4.41	1.17684E-49	Down	5.38077E-53
103644300	16.86	366.10	-4.44	1.43613E-57	Down	5.74551E-61
103639989	2.34	51.46	-4.46	6.90508E-13	Down	3.15715E-15
100382499	65.56	1444.57	-4.46	7.46482E-66	Down	1.49322E-69
103630514	6.00	132.30	-4.46	1.02643E-27	Down	1.34925E-30
100501307	2.38	55.42	-4.54	3.26644E-12	Down	1.55883E-14
100276889	13.68	334.44	-4.61	2.10382E-11	Down	1.11221E-13
100383808	14.41	354.13	-4.62	2.44067E-13	Down	1.06013E-15
100383090	43.32	1113.70	-4.68	1.29483E-16	Down	3.95917E-19
100274602	48.39	1294.16	-4.74	3.69282E-15	Down	1.3613E-17
100279365	2.35	66.64	-4.83	2.2368E-14	Down	8.75697E-17
103653182	6.06	173.34	-4.84	5.56978E-30	Down	6.36655E-33
100284209	4.44	135.27	-4.93	3.25731E-18	Down	8.7497E-21
100382026	4.96	157.90	-4.99	3.36525E-21	Down	7.40481E-24
100283756	17.33	560.89	-5.02	2.26843E-16	Down	7.06574E-19

100279509	28.26	930.43	-5.04	1.07716E-22	Down	2.00079E-25
100284097	20.63	685.42	-5.05	4.92648E-14	Down	1.97093E-16
100277247	187.83	6396.28	-5.09	2.96349E-90	Down	1.69372E-94
103639382	5.37	198.96	-5.21	1.6085E-30	Down	1.74667E-33
100283407	12.64	492.66	-5.28	3.89719E-18	Down	1.05799E-20
100501546	153.18	6065.96	-5.31	2.85681E-26	Down	4.16349E-29
100279972	77.28	3084.87	-5.32	3.97964E-29	Down	4.77639E-32
103628940	5.93	261.50	-5.46	5.559E-35	Down	5.08339E-38
100283034	16.54	807.50	-5.61	3.67766E-69	Down	6.30564E-73
542761	5.49	300.04	-5.77	5.66097E-30	Down	6.63256E-33
103643917	2.36	132.76	-5.81	6.16797E-24	Down	1.03992E-26
103640085	43.10	2526.49	-5.87	1.1824E-21	Down	2.399E-24
100383115	316.24	20193.11	-6.00	1.69054E-23	Down	2.94688E-26
100276192	29.01	1870.15	-6.01	1.8213E-31	Down	1.9257E-34
103637446	2.14	143.75	-6.07	1.10207E-27	Down	1.48017E-30
100282493	25.26	1718.40	-6.09	6.16754E-26	Down	9.34103E-29
100282491	262.82	20543.66	-6.29	2.52286E-27	Down	3.46052E-30
103646055	28.91	2559.80	-6.47	7.37075E-28	Down	9.4783E-31
100191623	49.96	6137.31	-6.94	4.09258E-58	Down	1.52036E-61
100274426	18.47	2292.44	-6.96	3.24332E-32	Down	3.24387E-35
100286025	41.64	5924.89	-7.15	3.54835E-39	Down	2.41093E-42
103643621	3.43	502.93	-7.20	1.16528E-49	Down	4.99492E-53
100502093	5.17	807.61	-7.29	2.93366E-44	Down	1.59283E-47
103648444	7.37	1579.30	-7.74	2.80627E-59	Down	9.62316E-63
100272446	2.72	769.96	-8.15	1.70432E-70	Down	2.43515E-74
103655377	4.47	1551.66	-8.44	9.78004E-78	Down	8.38433E-82
100191533	6.07	2141.97	-8.46	1.30806E-64	Down	2.99037E-68
